# Overexpression miR-125a-5p inhibits HSCs activation and alleviates liver fibrosis through TGF-β/Smad2/3 signaling pathway and autophagy

**DOI:** 10.1038/s41420-025-02694-4

**Published:** 2025-09-01

**Authors:** Chunyan Zhang, Yabin Zhao, Haoyu Yan, Jianlin Guo, GuoYing Yu

**Affiliations:** State Key Laboratory Cell Differentiation and Regulation, Henan International Joint Laboratory of Pulmonary Fibrosis, Henan Center for Outstanding Overseas Scientists of Pulmonary Fibrosis, College of Life Science, Institute of Biomedical Science, Henan Normal University, Xinxiang, Henan China

**Keywords:** Endocrine system and metabolic diseases, Cell signalling

## Abstract

Liver fibrosis represents an important pathological stage during chronic hepatopathy development, posing a significant threat to human health. Hepatic stellate cells (HSCs), an essential hepatic non-parenchymal cells, have a key effect on fibrogenesis, with their activation being a hallmark of liver fibrosis. MicroRNAs (miRNAs), the small non-coding RNAs, become the critical biomarkers and regulatory molecules in fibrotic processes. Among them, miR-125a-5p is implicated in cancer and inflammatory pathways, yet its functional role and mechanistic involvement in HSC activation remain poorly understood. According to our findings, miR-125a-5p expression was significantly decreased in TGF-β-activated HSC-T6 cells. Notably, ectopic miR-125a-5p overexpression effectively inhibited TGF-β-mediated HSC-T6 activation. Further mechanistic investigations revealed that miR-125a-5p attenuated HSC activation while ameliorating liver fibrosis through regulating the TGF-β/Smad2/3 pathway and autophagy. Additionally, TGFβR1 was miR-125a-5p’s target gene. Collectively, miR-125a-5p negatively regulates HSC activation in liver fibrosis, exerting its anti-fibrotic activities through suppressing the TGF-β/Smad2/3 pathway and autophagy modulation.

## Introduction

In recent years, liver disease has seriously threatened people’s health [1]. Liver fibrosis is the critical pathological transition during chronic hepatic disorder development. During this process, hepatocytes are repeatedly damaged and regenerated, and the extracellular matrix (ECM) is excessively deposited and distributed abnormally [2]. HSC activation represents a pivotal event in liver fibrosis pathogenesis. Upon activation, HSCs are the major source of ECM and exhibit high expression of α-smooth muscle actin (α-SMA), a key mediator in the progression of fibrotic liver disease [3]. Activated HSCs are also the main source of collagen in the liver, and can secrete a large number of metalloproteinase inhibitors, extracellular matrix proteins, etc., thus triggering the reconstruction of liver structure [4–6]. In addition, liver fibrosis is a key contributor to long-term morbidity (such as cirrhosis or liver cancer) in patients with nonalcoholic steatohepatitis (NASH) or nonalcoholic fatty liver disease (NAFLD) [7]. If liver fibrosis is not treated in time, it may further progress to cirrhosis and even liver cancer [5]. However, until now, the regulatory mechanisms of HSC activation have not been fully understood. Consequently, studying the HSC activation mechanisms is important to prevent and treat liver fibrosis.

miRNAs, the endogenous small non-coding RNAs, are usually 21–22 nucleotides long, which exert crucial effects on post-transcriptional gene regulation. Through combining with 3′ untranslated region (3′UTR) in target mRNAs, miRNAs modulate intracellular and extracellular signaling pathways through epigenetic mechanisms [8, 9]. miRNAs are important for various liver pathologies, where they modulate hepatic inflammatory responses and certain gene and protein levels in liver fibrosis [10, 11]. Some miRNAs, like miR-488 [12], miR-340 [13], miR-17-5p [14], exert critical effects on modulating HSC activation, growth and apoptosis. miR-125a-5p is a highly conserved miRNA with down-regulated expression in a variety of cancers and is a cancer-suppressing miRNA [15–18]. miR-125a-5p is implicated in pathogenic mechanisms of inflammatory disorders, like diabetic retinopathy, multiple sclerosis, and atherosclerosis, as a new modulator of Ninjurin1, the adhesion molecule that modulates the activity of macrophages in vitreous degeneration, multiple sclerosis and atherosclerosis [19]. In the myocardial ischemia/reperfusion (I/R) injury murine model, administering miR-125a-5p agomir enhanced M2 macrophage polarization, stimulated angiogenesis, suppressed fibroblast proliferation and activation, and thus improved cardiomyocyte apoptosis and inflammation [20]. For chronic hepatitis B (CHB) individuals, miR-125a-5p is implicated in modulating hepatitis B virus (HBV) replication and contributing to disease development [21]. miR-125a-5p also predicts human liver disease progression [22]. Nevertheless, the functional significance and mechanistic basis underlying miR-125a-5p during HSC-driven liver fibrosis remain incompletely understood and warrant further exploration.

According to our findings, miR-125a-5p expression decreased in activated HSC-T6 cells, while ectopic miR-125a-5p expression suppressed HSC-T6 cell activation. As revealed by the mechanism study result, miR-125a-5p suppressed HSC-T6 cell activation via the TGF-β/Smad2/3 pathway and autophagy. The above results position miR-125a-5p as the novel regulatory element of the TGF-β pathway within HSCs during fibrogenesis, underscoring its role as the therapeutic target for liver fibrosis intervention.

## Results

### miRNA-125a-5p was decreased in activated HSC-T6 cells

**Fig. 1:**
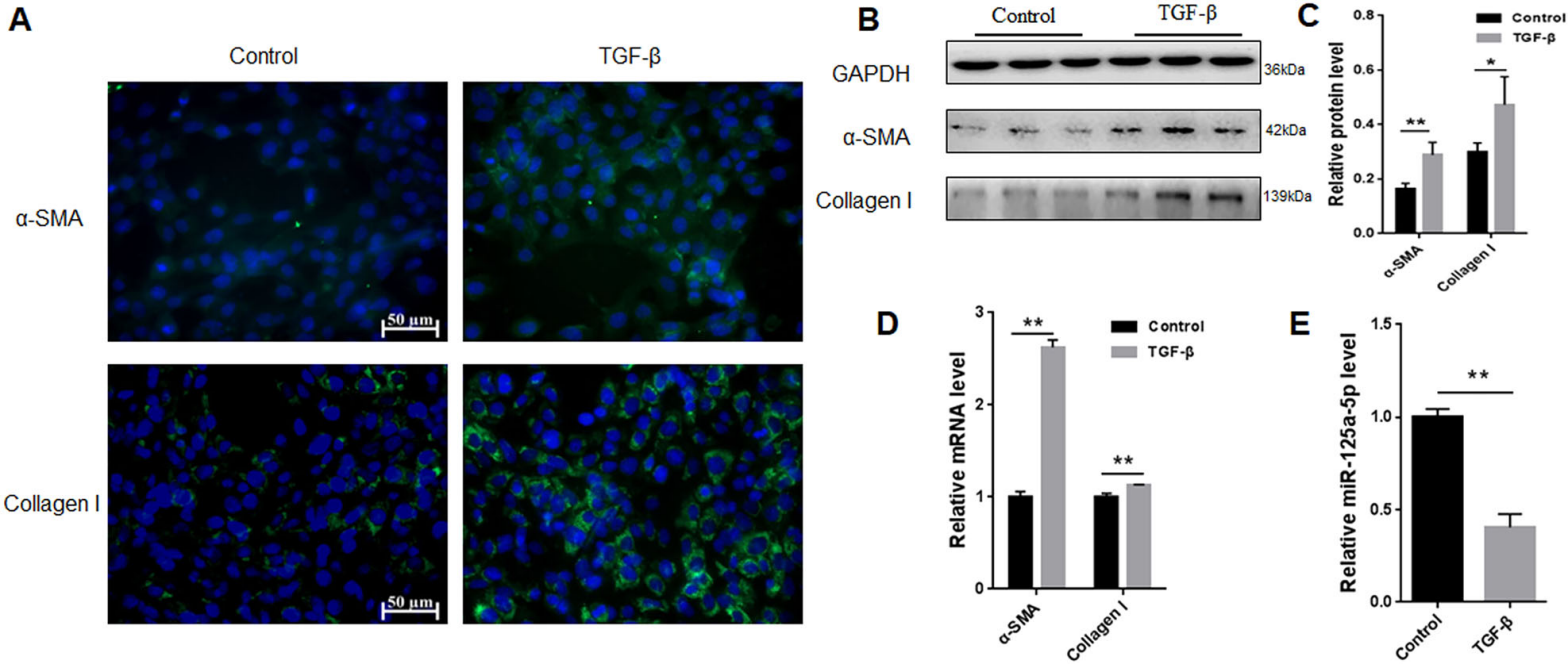
miRNA-125a-5p level in activated HSC-T6. **A** Immunofluorescence was carried out for evaluating α-SMA and Collagen I levels, **B** WB was conducted to evaluate α-SMA and Collagen I protein levels, **C** Quantitative densitometry for analyzing relative α-SMA and Collagen I levels from Western blot data, **D** qRT-PCR was employed for measuring α-SMA and Collagen I mRNA expression, **E** qRT-PCR was conducted for quantifying miRNA-125a-5p expression. *p < 0.05, ** *p* < 0.01.

Previously, miRNA levels were associated with HSCs activation [23]. For analyzing how miR-125a-5p affected HSCs activation, we established an in vitro model of TGF-β-mediated activation of HSC-T6 cells and examined miR-125a-5p expression under these conditions. First, we detected fibrosis markers and found that α-SMA and Collagen I were up-regulated within activated HSC-T6 cells relative to controls (Fig. 1A–D), indicating that this TGF-β-mediated HSC-T6 cell activation model was successfully constructed. Relative to control, miR-125a-5p expression was decreased in activated HSC-T6 (Fig. 1E). Consequently, miRNA-125a-5p may inhibit TGF-β-mediated HSC-T6 activation.

### Overexpression of miRNA-125a-5p inhibited TGF-β-induced HSC-T6 cells activation

**Fig. 2:**
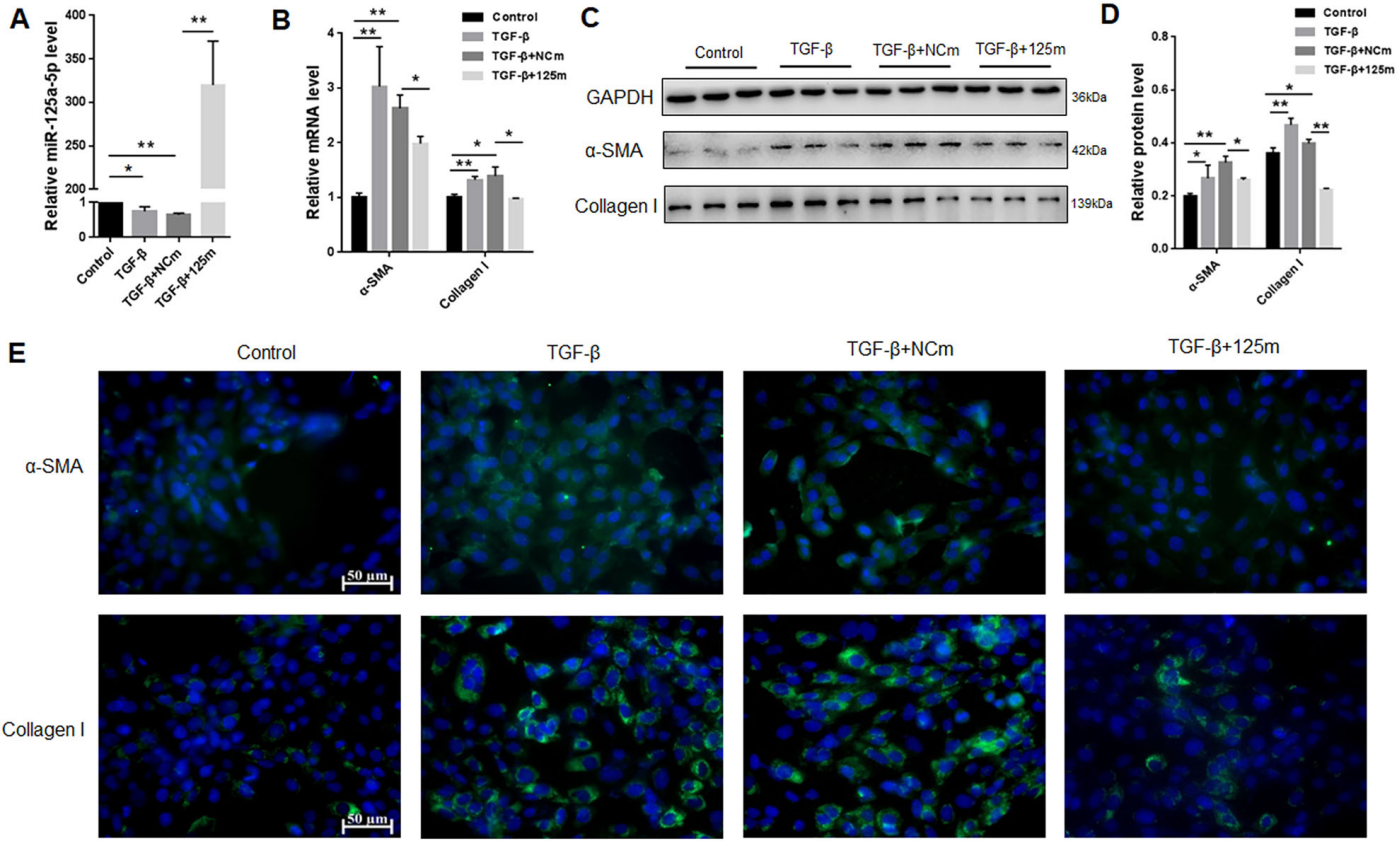
Role of miRNA-125a-5p overexpression in activated HSC-T6 cells. **A** qRT-PCR was performed for assessing miRNA-125a-5p expression, **B** qRT-PCR was conducted for evaluating α-SMA and Collagen I expression, **C** WB was performed for detecting α-SMA and Collagen I protein levels, **D** quantification of α-SMA and Collagen I protein expression, **E** immunofluorescence was employed to measure the α-SMA and Collagen I levels. *p < 0.05, ** *p* < 0.01.

To examine the effect of miRNA-125a-5p on TGF-β-induced HSC-T6 cells activation, we transfected miRNA-125a-5p mimics (125 m) and NC mimics (NCm) in TGF-β-activated HSC-T6 cells. We first examined the expression of miRNA-125a-5p in each group. Compared with the control group, miRNA-125a-5p expression was downregulated in TGF-β treatment and TGF-β + NCm groups (Fig. 2A). Compared with TGF-β + NCm group, miRNA-125a-5p expression of TGF-β + 125 m group was up-regulated (Fig. 2A). Subsequently, we examined the fibrosis markers and found that α-SMA and Collagen I levels of TGF-β-activated HSC-T6 cells were up-regulated, but the up-regulation of α-SMA and Collagen I was suppressed after 125 m transfection (Fig. 2B–E). Consequently, miRNA-125a-5p may inhibit TGF-β-mediated HSC-T6 cell activation.

### miRNA-125a-5p overexpression activated autophagy in activated HSC-T6 cells

**Fig. 3:**
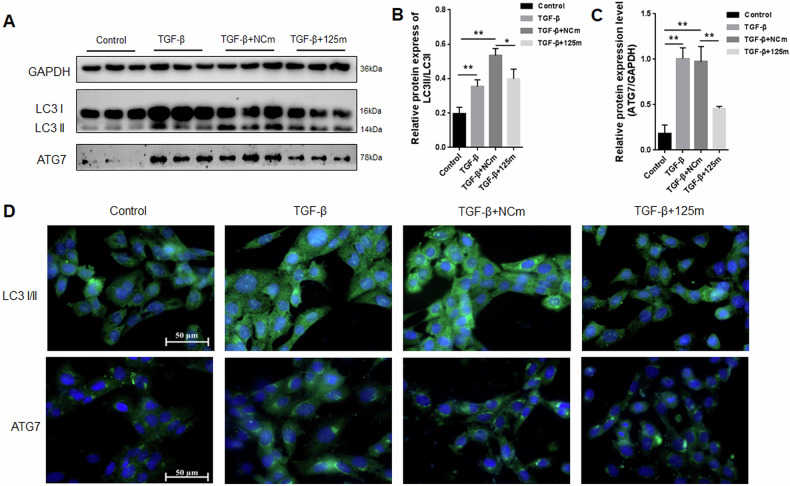
Function of miRNA-125a-5p overexpression in the activated HSC-T6 cell autophagy. **A** WB was conducted for evaluating LC3 and ATG7 protein levels, **B** quantification of LC3I/LC3II protein expression, **C** quantitative analysis of ATG7 protein expression, **D** immunofluorescence was employed to measure LC3 and ATG7 expression. * *p* < 0.05, ** *p* < 0.01.

Numerous studies have demonstrated a close association between HSCs activation and autophagy [24, 25]. To determine whether miRNA-125a-5p modulates the activated HSC-T6 cell autophagy, autophagy markers LC3 and ATG7 were explored inside activated HSC-T6 cells. Compared with the control group, LC3 and ATG7 expression were markedly upregulated after TGF-β treatment, but the up-regulated expressions of LC3 and ATG7 were inhibited through overexpressing miR-125a-5p (Fig. 3A–D). Based on the above findings, miRNA-125a-5p overexpression attenuates the TGF-β-mediated HSC-T6 cell activation, potentially mediated through inducing autophagy.

### miRNA-125a-5p inhibited HSC-T6 cells activation through TGF-β/Smad2/3 pathway

**Fig. 4:**
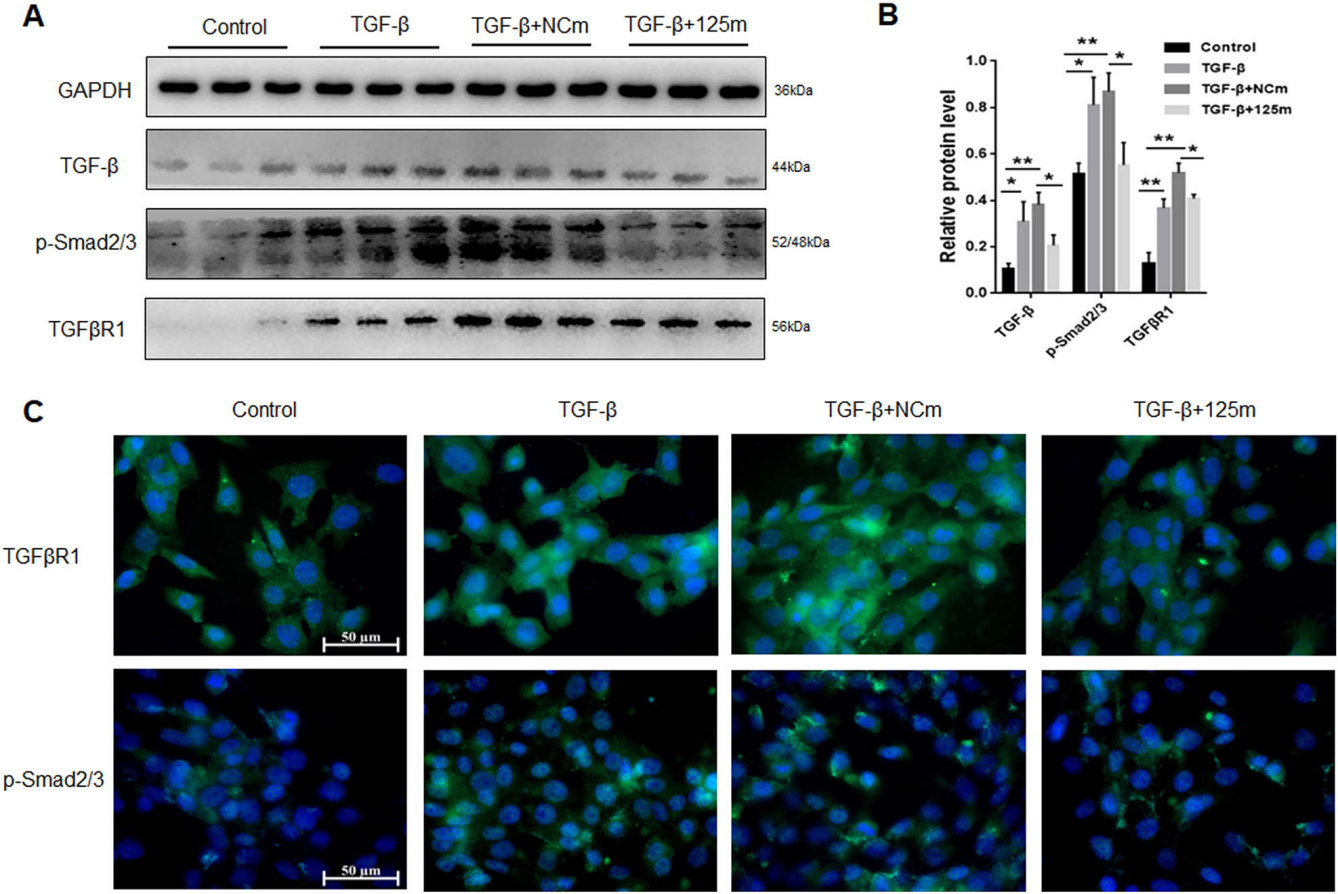
Function of miRNA-125a-5p overexpression in TGF-β/Smad pathway of activated HSC-T6 cells. **A** WB was conducted to estimate TGF-β, TGFβR1 and p-Smad2/3 levels, **B** quantitative analysis of TGF-β, TGFβR1 and p-Smad2/3 protein levels, **C** immunofluorescence was employed to measure the expression of TGFβR1 and p-Smad2/3. **p < 0.05, ** *p* < 0.01.

TGF-β/Smad is a classical signaling pathway for HSCs activation [26]. In order to verify whether miRNA-125a-5p affects HSC-T6 activation via TGF-β/Smad pathway. We detected the changes in some key gene expression in the TGF-β/Smad2/3 pathway. As discovered, TGF-β, TGFβR1 and p-Smad2/3 were up-regulated following TGF-β treatment. miR-125a-5p overexpression inhibited upregulated TGF-β level, TGFβR1 and p-Smad2/3 (Fig. 4A–C). These findings indicated that miRNA-125a-5p can suppress HSC-T6 cell activation by TGF-β/Smad pathway.

### TGFβR1 was the target gene of miRNA-125a-5p

**Fig. 5:**
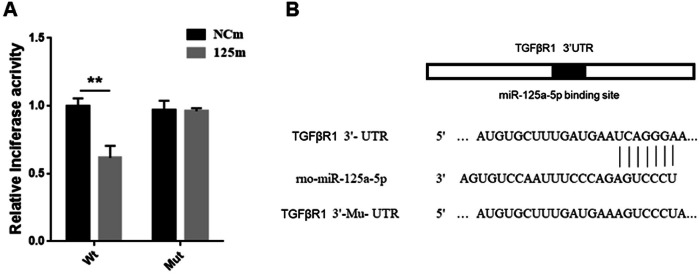
TGFβR1 is miR-125a-5p’s target. **A** Luciferase reporter assays were performed in HSC-T6 cells after TGFβR1- WT/Mu-UTR constructs and miR-125a-5p mimics or negative control (NC) mimics were co-transfected in these cells. **B** The miR-125a-5p estimated binding site with TGFβR1-WT/Mu-UTR. Results represent mean ± SEM, ** *p* < 0.01.

Through integrated bioinformatic analysis using miRDB (https://mirdb.org/cgi-bin/search.cgi), TargetScan (https://www.targetscan.org/vert_80/), and other prediction tools, along with prior literature evidence [20], we identified TGFβR1 as a putative target gene of miRNA-125a-5p. To validate this interaction, we performed dual-luciferase reporter assays in HSC-T6. These results demonstrated that luciferase activity driven by wild-type TGFβR1 3′-UTR significantly decreased upon transfection with miRNA-125a-5p mimics. But obvious changes were not detected when the 3′-UTR contained mutations (Fig. 5A, B). These findings conclusively established TGFβR1 as the miRNA-125a-5p’s direct target in this cellular context.

### miR-125a-5p overexpression inhibits TGFβR1 in vitro and in vivo

**Fig. 6:**
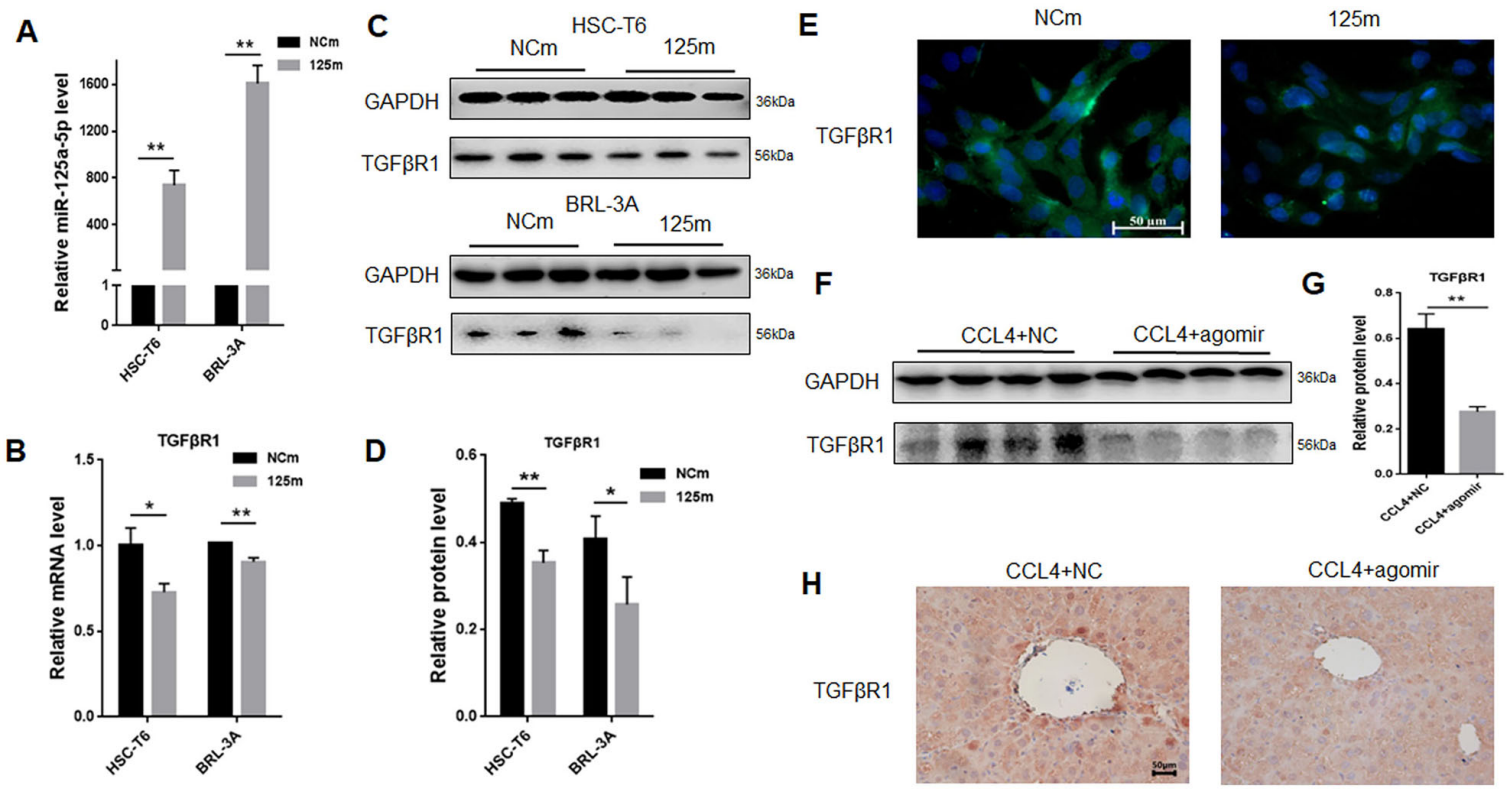
miR-125a-5p overexpression inhibits TGFβR1 in vitro and in vivo. **A** qRT-PCR was employed for analyzing miRNA-125a-5p expression within HSC-T6 and BRL-3A cells overexpressing miRNA-125a-5p, **B** qRT-PCR was conducted for evaluating TGFβR1 expression within both cell lines overexpressing miRNA-125a-5p, **C** WB was performed for exploring TGFβR1 level in both cell lines overexpressing miRNA-125a-5p, **D** quantitative analysis TGFβR1 protein level in both cell lines overexpressing miRNA-125a-5p, **E** immunofluorescence was used to evaluate TGFβR1 expression in HSC-T6 cells overexpressing miRNA-125a-5p, **F** WB was conducted detect TGFβR1 expression in mouse liver tissues overexpressing miRNA-125a-5p, **G** quantitative analysis TGFβR1 protein levels inside liver tissues in liver fibrosis mice overexpressing miRNA-125a-5p, **H** immunohistochemistry was employed to evaluate TGFβR1 expression inside liver tissues in liver fibrosis mice overexpressing miRNA-125a-5p. *p < 0.05, ** *p* < 0.01.

MiRNAs work by targeting mRNA, causing it to degrade or inhibiting its transcription. Therefore, we transfected 125 m in HSC-T6 cells and BRL-3A cells, respectively. As discovered, TGFβR1 had decreased mRNA and protein levels in the 125 m group compared with NCm (Fig. 6A–E). Meanwhile, we also verified in vivo. Compared to the agomir NC (NC) group, TGFβR1 expression in the fibrotic mice liver was apparently decreased in the miRNA-125a-5p agomir (agomir) group (Fig. 6F–H). We indirectly demonstrated TGFβR1 as miRNA-125a-5p’s target gene.

### Construction and identification of a mouse liver fibrosis model

**Fig. 7:**
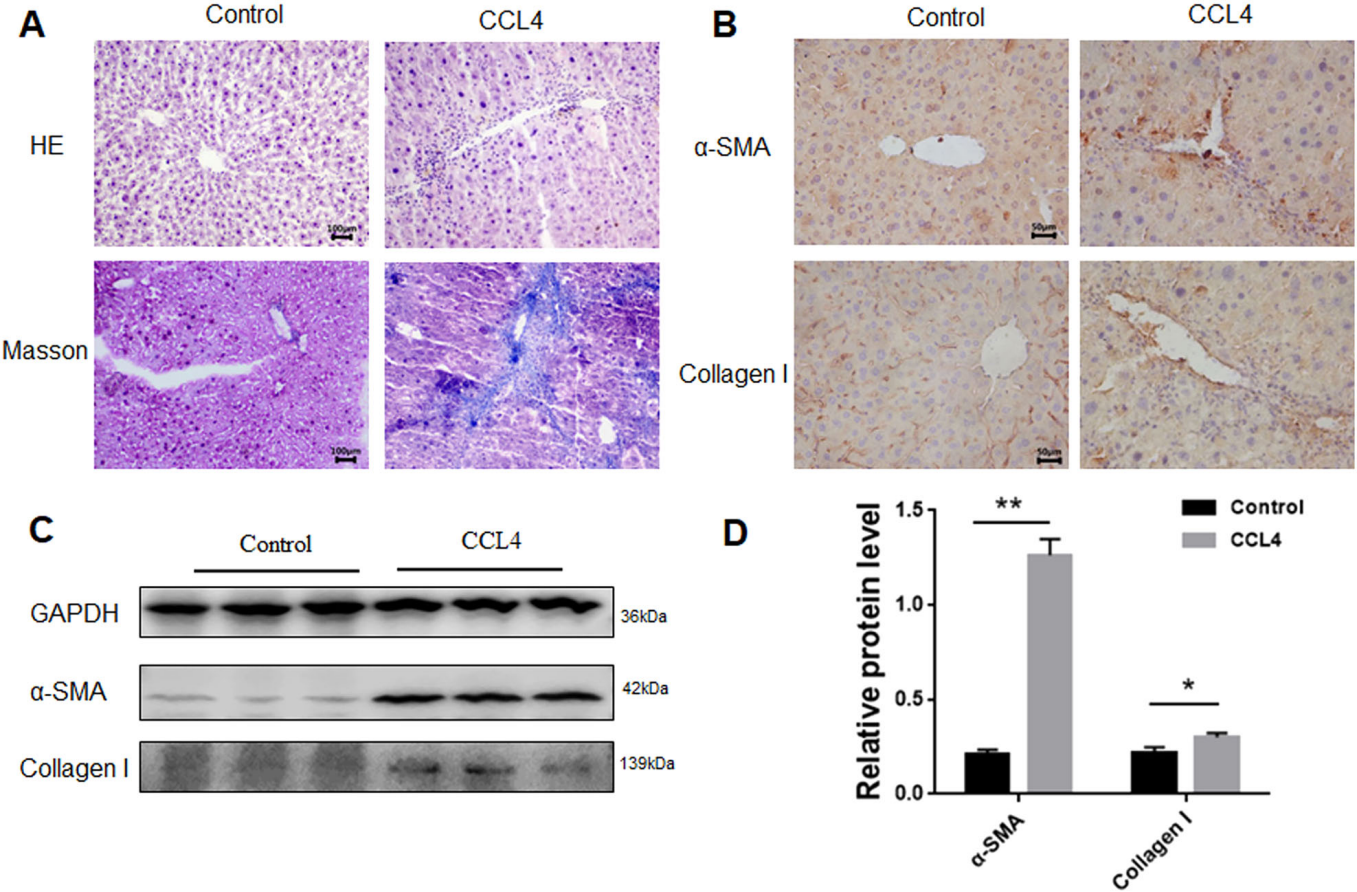
Detection of fibrosis indices in the liver tissue of fibrotic mice. **A** HE and Masson assay were used to detect liver tissue of mice with fibrosis, **B** immunohistochemistry was used to detect α-SMA and Collagen I levels in fibrotic liver tissues, **C** WB was performed to detect α-SMA and Collagen I levels inside fibrotic liver tissues, **D** quantitative analysis of α-SMA and Collagen I protein expression within fibrotic liver tissues. *p < 0.05, ** *p* < 0.01.

CCL_4_-mediated mouse liver fibrosis model is a commonly used model for studying liver fibrosis [27, 28]. For analyzing how miRNA-125a-5p affected mouse liver fibrosis, this study constructed the CCL4-mediated mouse liver fibrosis model. Firstly, fibrosis indices were detected to determine whether the liver fibrosis model was successfully constructed. Relative to the control group, HE results showed that inflammatory cells were apparently elevated and liver tissue structure was markedly broken in the liver of fibrotic mice induced by CCL4 (Fig. 7A), and Masson staining showed that collagen deposition was remarkably elevated within the liver in fibrotic mice (Fig. 7A). As revealed by WB and IHC findings, fibrosis markers α-SMA and Collagen I were significantly increased (Fig. 7B–D). These results confirmed the successful establishment of a CCl_4_-induced liver fibrosis murine model.

### miRNA-125a-5p Agomir expression in the fibrotic mouse model

**Fig. 8:**
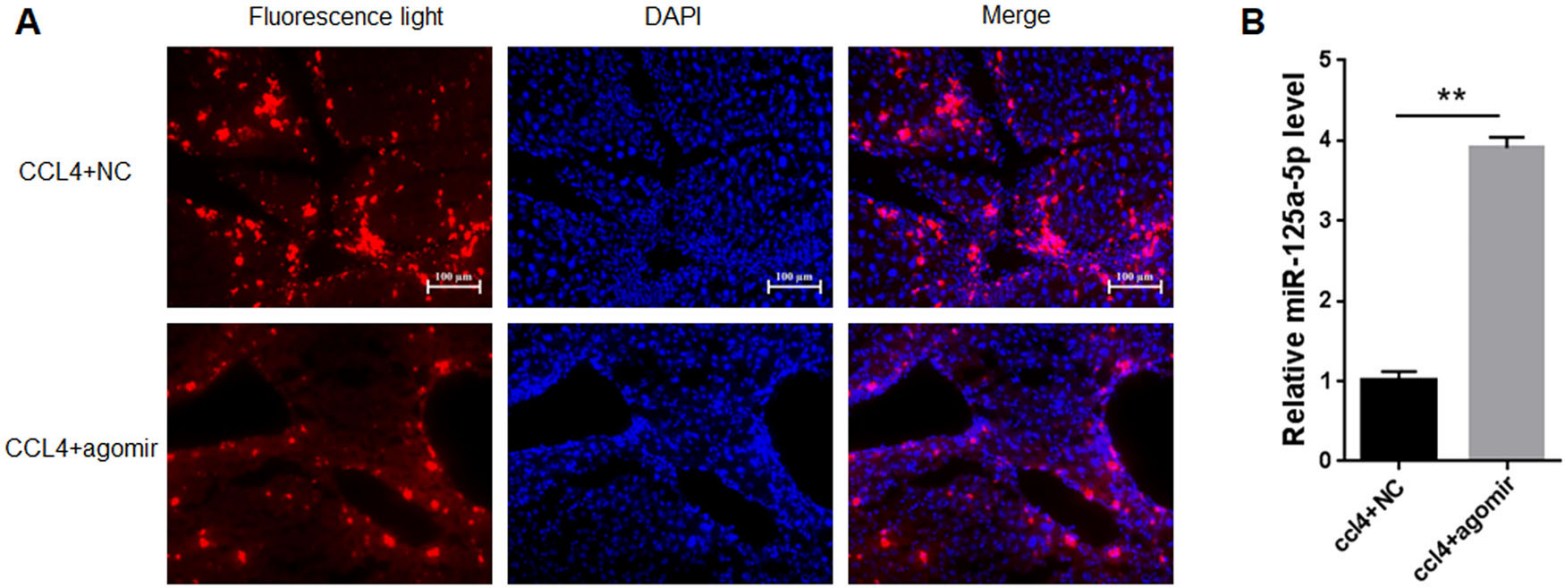
Transfection efficiency of miRNA-125a-5p agomir in fibrotic liver tissues. **A** Fluorescence microscope was employed for detecting the miRNA-125a-5p agomir transfection efficiency in mice with liver fibrosis, **B** qRT-PCR was carried out for evaluating the miRNA-125a-5p agomir transfection efficiency in mice with liver fibrosis. ** *p* < 0.01.

For investigating how miR-125a-5p affected murine liver fibrosis, miRNA-125a-5p agomir(agomir) and its control NC(NC) were administered via tail vein injection in a well-established liver fibrosis mouse model, and then miRNA-125a-5p expression within liver tissues was analyzed. Fluorescence observation showed that CY-5 labeled cells were significantly increased in liver tissue (Fig. 8A), and qRT-PCR analysis demonstrated a significant upregulation of miR-125a-5p expression following administration of agomir (Fig. 8B). These results indicated that the mouse liver fibrosis model with miRNA-125a-5p overexpression has been successfully established.

### Roles of miRNA-125a-5p in mouse liver fibrosis

**Fig. 9:**
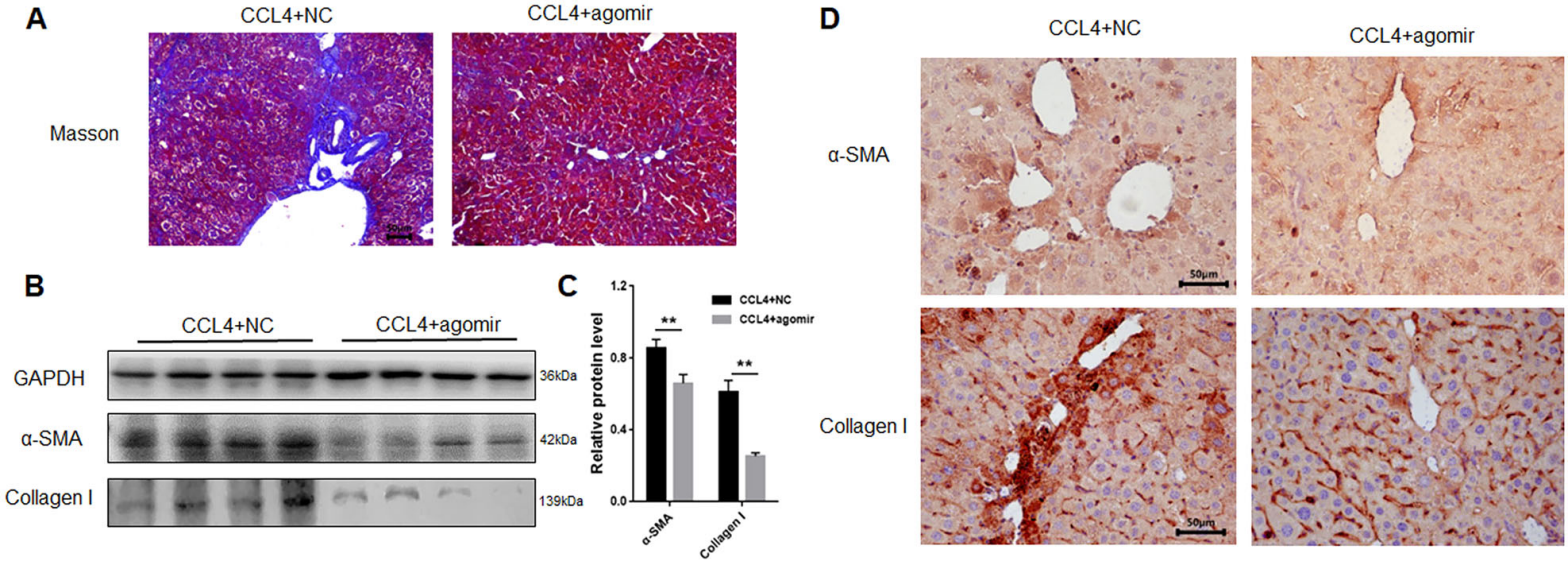
Assessment of fibrosis indexes in mice liver fibrosis overexpressing miRNA-125a-5p. **A** Masson assay was used to appraise collagen deposition in fibrotic liver tissues overexpressing miRNA-125a-5p, **B** WB was conducted to evaluate the α-SMA and Collagen I levels within fibrotic liver tissues overexpressing miRNA-125a-5p, **C** Quantitative analysis on α-SMA and CollagenI protein levels inside liver tissues of mice with liver fibrosis overexpressing miRNA-125a-5p, **D** immunohistochemistry was used to detect the α-SMA and Collagen I expression within fibrotic mouse liver tissues overexpressing miRNA-125a-5p. ** *p* < 0.01.

We examined the fibrosis indexes in mouse liver tissues overexpressing miRNA-125a-5p. Compared with the NC group, Masson staining revealed significantly reduced collagen deposition in the mouse liver overexpressing miRNA-125a-5p (Fig. 9A), and α-SMA and Collagen I expression (Fig. 9B–D) were significantly decreased in the mouse liver overexpressing miRNA-125a-5p. Therefore, overexpression of miRNA-125a-5p alleviates liver fibrosis progression.

### miRNA-125a-5p alleviates mouse liver fibrosis via TGF-β/Smad2/3 axis and autophagy

**Fig. 10:**
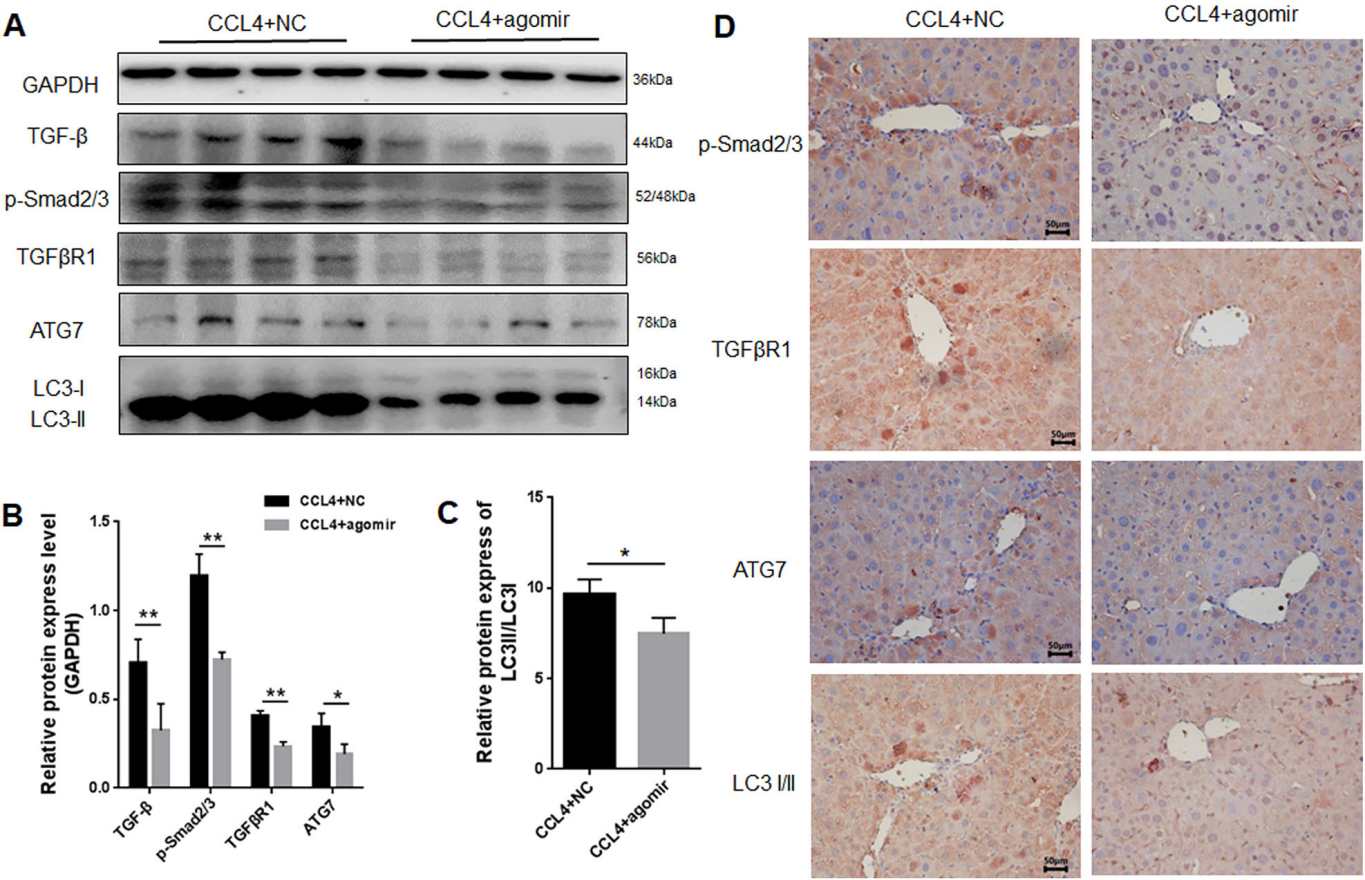
Detection of key proteins of autophagy and TGF-β/Smad signaling pathways in the liver fibrosis of mice overexpressing miRNA-125a-5p. **A** WB was conducted to evaluate the key proteins of autophagy and TGF-β/Smad pathway in mouse liver tissues overexpressing miRNA-125a-5p, **B** quantitative analysis the key proteins of autophagy and TGF-β/Smad pathway in fibrotic mouse liver tissues overexpressing miRNA-125a-5p, **C** quantification of LC3I/LC3II protein level in mouse liver tissues overexpressing miRNA-125a-5p, **D** IHC was used to detect the key proteins of autophagy and TGF-β/Smad pathway in fibrotic mouse liver tissues overexpressing miRNA-125a-5p. *p < 0.05, ** *p* < 0.01.

To reveal the mechanism of miRNA-125a-5p alleviating mouse liver fibrosis, key proteins in the TGF-β/Smad pathway and autophagy-related proteins in mouse liver tissues overexpressing miRNA-125a-5p were detected. In comparison with the NC group, WB and IHC results revealed the inhibited expressions of autophagy-related proteins LC3 and ATG7 in the fibrotic mice after 125 m transfection, and the expressions of key proteins in TGF-β/smad pathway, TGFβR1 and p-Smad2/3, were also inhibited (Fig. 10A–D). These results suggested that, similar to the results in vitro, miRNA-125a-5p can inhibit the progression of CCL4-induced liver fibrosis in mice by modulating the TGF-β/Smad signaling pathway and autophagy.

## Discussion

According to this work, miR-125a-5p is related to HSCs activation during fibrosis. From a mechanism perspective, miR-125a-5p can act as a new TGF-β inhibitor in HSC activation and may be a molecular and/or biomarker for fibrosis therapy. miR-125a-5p expression was markedly reduced within activated HSC-T6 cells. Forced mature miR-125a-5p overexpression inside activated HSC-T6 cells significantly reduced TGFβR1 expression, promoted autophagy related proteins LC3 and ATG7, and effectively inhibited the expression of fibrosis genes, conforming to relevant literature that the activation of autophagy can inhibit fibrosis progression [24, 29] In addition, by observing the activation phenotype of HSCs, miR-125a-5p overexpression significantly suppressed HSC-T6 cells activation marker α-SMA expression and the deposition of Collagen 1. These results align with existing literature, which confirms that HSC activation can be assessed through alterations in transdifferentiation marker expression (reduced α-SMA and Collagen 1) [30–32]. These results broaden the miRNA research scope in liver fibrosis and establish a theoretical foundation for targeted therapeutic interventions.

miRNAs have been established as pivotal regulatory molecules in the pathogenic mechanism of chronic hepatopathy (like liver fibrosis) [10] and are involved in HSCs activation in liver fibrosis [14]. For example, miR-301a-3p expression is elevated in liver fibrosis patients, which activates human HSCs (LX-2), while inhibition of miR-301a-3p can alleviate mouse liver fibrosis and collagen deposition, and inhibit HSCs activation and fibrotic factor expression [33]. Extracellular vesicles rich in miR-181a-5p drive hepatic metastasis through hepatic stellate cell activation and tumor microenvironment remodeling [34]. Exosomal miRNA-21 derived from hepatocellular carcinoma has been shown to facilitate tumor progression by transforming HSCs into cancer-associated fibroblasts [35]. These findings underscore the critical effects of miRNAs on activating HSCs and the liver fibrosis pathogenesis, highlighting their potential as therapeutic targets for fibrotic liver disease. miR-125a-5p overexpression inside activated HSC-T6 cells inhibited type I collagen and α-SMA expression of HSC-T6 cells.

Regardless of the varying pathologies of chronic liver disease (ALD, HCV, HCC), they share a common underlying mechanism: TGFβ-mediated fibrosis [36]. TGFβ is the most potent stimulator of HSCs-mediated fiber formation [37, 38], primarily due to its pivotal role in initiating transdifferentiation. Beyond paracrine cytokine signaling, activated HSCs exhibit a marked upregulation in TGF-β synthesis, thereby reinforcing the fibrotic phenotype. Inhibition of TGFβ receptors, specifically TGFβR1, blocks TGFβ/Smad2/3 signaling pathways and HSCs activation [39–41]. We also demonstrated that miR-125a-5p overexpression effectively suppresses expression of TGFβR1 and p-Smad2/3 in both activated HSC-T6 cells and fibrotic liver tissues in mice. miRNAs function mainly through their target genes. Previous studies have found that TGFβR1 is the target of miR-125a-5p [20]. Using a dual-luciferase reporter assay, as well as in vitro and in vivo experiment results, TGFβR1 is indeed miR-125a-5p’s target, and overexpressing miR-125a-5p effectively suppresses TGFβR1 expression.

Autophagy has an important effect on regulating liver homeostasis, and the dysregulated autophagy is associated with the pathogenic mechanisms of diverse chronic liver disorders [42, 43]. Notably, the functional impact of autophagy in liver disease exhibits both cell-type specificity and disease-stage dependence [43–45]. In liver fibrosis, HSCs activation is induced by the degradation of lipid droplets, known as lipophagy [46]. In hepatocytes, autophagy has been characterized as an anti-fibrotic mechanism, as it promotes hepatocyte survival signaling pathways that mitigate fibrotic progression [43]. Recently, inhibiting autophagy in hepatocytes was associated with increased extracellular vesicles (EVs) release in alcoholic liver disease [47]. Gao et al. found that HSCs autophagy inhibited the release of extracellular vesicles and alleviated liver fibrosis [48]. Zhang *et al*. found that destruction of the TRIB3-SQSTM1 interaction could reduce the degree of liver fibrosis by restoring autophagy in the BDL model [24]. Also, miR-125a-5p overexpression suppressed LC3 and ATG7 in activated HSC-T6 and the CCL4-injured liver tissues. It has been demonstrated that autophagy alleviates liver fibrosis through suppressing HSCs activation, and is one of the potential targets for future fibrosis therapy.

Collectively, miR-125a-5p can inhibit HSCs activation and alleviate liver fibrosis to some extent through the TGF-β/Smad2/3 signaling pathway and autophagy. It can be used as the new modulator for the TGF-β pathway in fibrosis and is a possible therapy to treat or reverse fibrosis. Our findings shed new light on the global regulation of key signaling pathways during liver fibrosis, and more importantly, provide a new way for exploring HSC transformation in liver fibrosis.

## Materials and methods

### Liver fibrosis model and tail vein injection

Adult Kunming mice (male, weighing 25 $\pm$ 5 g) were acquired from the laboratory animal control office of Henan Normal University. Animal experimental protocols gained approval from the Institutional Animal Care and Use Committee (IACUC) of Henan Normal University (License number: HNSD-2021-07-12), conducted in strict compliance with the Animal Protection Law. In short, altogether 24 mice were randomized into four experimental groups(*n* = 6). They were allocated into the control group, the carbon tetrachloride (CCL4) group, the CCL4 + miRNA-125a-5p agomir group and the CCL4 + NC agomir group. The preparation of a mouse model of liver fibrosis was induced by CCL4 dissolved in olive oil for a final concentration of 20%, and intraperitoneally injected 1 ml/kg twice a week for six weeks. At the sixth week, miRNA-125a-5p agomir or NC agomir (miR40000829-4-5, Ribobio) were injected into the mice through the tail vein, once every 2 days, three times.

### Fluorescence observation

The mice liver tissues were obtained and made into frozen sections according to the previously published paper [8], in brief, the liver tissues were processed through embedding in frozen section embedding agent and then cutting into 5 μm thick slices. After 4% paraformaldehyde fixation, DAPI was added for 10 min of nuclear staining, and a positive fluorescence microscope (Axio Imager D2, Carl Zeiss, Germany) was utilized to observe the sections and take photos.

### Cell grouping and treatment

HSC-T6 cells (CL-0116, Procell) were classified as four groups following as control group, TGF-β (80116-RNAH, SinoBiological) treatment group (48 h of 5 ng/ml TGF-β treatment), TGF-β+mimic NC group (24 h of 5 ng/ml TGF-β treatment and then 24 h of mimic NC transfection), and TGF-β + miR-125a-5p group(5 ng/ml TGF-β for 24 h, and transfected miR-125a-5p mimic (miR10000829-1-5, Ribobio) for 24 h.) Lipo RNAi MAX was used for transfection (13778-075, Thermo Fisher), and the method was carried out according to our previous paper [8]. The final miR-125a-5p mimic and mimic NC concentrations were 50 nM/L.

### Western Blot

By utilizing RIPA lysis buffer (Beyotime Biotechnology), proteins were extracted. Protein content was measured by BCA kit (Solarbio), and later separated through SDS-PAGE, prior to transfer onto the PVDF membrane. Different antibodies were used to incubate the membrane, including GAPDH (1:3000) (Affinity), α-SMA (1:1000) (Affinity), Collagen I (1:1000) (Affinity), LC3 (1: 1000) (Affinity), p-Smad2/3 (1:1000) (Biyuntian), ATG7 (1:1000) (Shanghai Bioengineering), TGFβR1 (1:1000) (Shanghai Bioengineering), TGF-β (1:1000) (Shanghai Bioengineering). The washed membrane was further incubated using horseradish peroxidase (HRP)-conjugated rabbit secondary antibody (1:5000, Affinity). An ECL chemiluminescence substrate was later added for protein band visualization, whereas ChemiDoc XRS System (Bio-Rad, USA) was applied in imaging. Image Lab Software (Bio-Rad) was employed for quantifying band intensities, and GAPDH was an endogenous reference for normalization.

### RNA isolation and qRT-PCR

Total cellular or tissue RNA was separated with TRIzol reagents and after the RNA concentration and purity were determined, the RNAs were reverse-transcribed in cDNA with the reverse transcription kit (Promega) following kit protocols. U6 or GAPDH was the endogenous reference, and the relative gene level was determined by 2^−^^ΔΔCt^ approach. Table 1 presents primer sequences used in the present work.

**Table 1:** Primers used in reverse transcription and quantitative real-time PCR.

miRNA and Genes	Pimers Sequences (5′ → 3′)
miRNA-125a-5p RT	GTCGTATCCAGTGCAGGGTCCGAGGTA
	TTCGCACTGGATACGACTCACAG
miRNA-125a-5p FP	TCCCTGAGACCCTTTAACCT
miRNA-125a-5p RP	GTGCAGGGTCCGAGGT
U6 FP	CTCGCTTCGGCAGCACA
U6 RP	AACGCTTCACGAATTTGCGT
Rat-GAPDH FP	AAGATGGTGAAGGTCGGTGTGA
Rat-GAPDH RP	TCGCTCCTGGAAGATGGTGAT
Rat-α-SMA FP	GTTGGAATGGGCCAAAAGGAC
Rat-α-SMA RP	CTCCGTTAGCAAGGTCGG
Rat-Collagen-I FP	CCTACAGCACGCTTGTGGATGG
Rat-Collagen-I RP	CAGATTGGGATGGAGGGAGTTTA
Rat-TGFβR1 FP	GAAATCGCTCGACGCTGTTC
Rat -TGFβR1 RP	TTCGCAAAGCTGTCAGCCTA

### HE and Masson

Mouse liver tissue specimens were subjected to 24 h of 4% paraformaldehyde fixation, then dehydration, paraffin embedding, and sectioning at 5 μm. After dewaxing, these slices underwent hematoxylin and eosin staining and were sealed with neutral gum. Masson staining was performed with Weigert hematoxylin, ponceau fuchsin and aniline blue. Detailed procedures were carried out according to the Masson dyeing kit (Solebel). Finally, a microscope was used to observe and photograph the stained slices.

### Immunohistochemistry

After dewaxing, 5 μm paraffin slices were sealed with endogenous peroxidase, and then hot repaired with sodium citrate buffer. After being sealed with 10% goat serum, the corresponding primary antibody (1:100) was incubated overnight at 4 °C, and later secondary antibody (1:500), DAB color rendering, dehydration, and neutral gum sealing tablets. Finally, a microscope is used to observe and photograph.

### Cellular immunofluorescence

After fixing the cell slides using 4% paraformaldehyde, they were subjected to permeabilization using 0.3% Triton, sealing with BSA, 18 h of primary antibody (1:100) incubation under 4 °C, rinsing by PBS, and 30 min of secondary antibody 1:500) incubation under 37 °C. DAPI was added for 10 min of nuclear staining. Finally, fluorescence microscope was used to observe and photograph.

### Luciferase vector acquisition and detection

First, the 3′UTR region in TGFβR1 that contained miRNA-125a-5p binding site was amplified before insertion into the psiCHECK-2 vector (Promega, Madison, WI, USA). In parallel, a mutant version of the TGFβR1 3′UTR lacking the miRNA-125a-5p recognition sequence was generated and cloned into this vector. Subsequently, HSC-T6 cells were inoculated and adhered overnight before co-transfection with miRNA-125a-5p mimics along with wild-type (WT) or mutant (Mut) reporter constructs. Finally, luciferase activity measurement was completed with a dual-luciferase assay kit (Promega, Madison, WI, USA).

### Data analysis

GraphPad Prism 6.0 software was applied in analyzing data represented by Mean $\pm$SEM. Between-group difference was analyzed with the independent *t*-test or ANOVA, containing the post hoc Tukey test. *P* < 0.05 stood for significant differences.

## Supplementary information

The original diagram of HE+Masson

The original diagram of IHC

The original diagram of immunofluorescence

The original gel diagram of WB

## Data Availability

All data supporting the findings of this study appear in the submitted manuscript or are available from the corresponding author upon reasonable request.
