# Supplementary material for: Overexpression miR-125a-5p inhibits HSCs activation and alleviates liver fibrosis through TGF-β/Smad2/3 signaling pathway and autophagy
Source: Cell Death Discov. 2025 Sep 1;11:419. doi: 10.1038/s41420-025-02694-4 (PMC12402229; doi:10.1038/s41420-025-02694-4)

Unedited original HE and Masson diagram for Figure 7A HE and Masson assay were used to detect liver tissue of mice with fibrosis

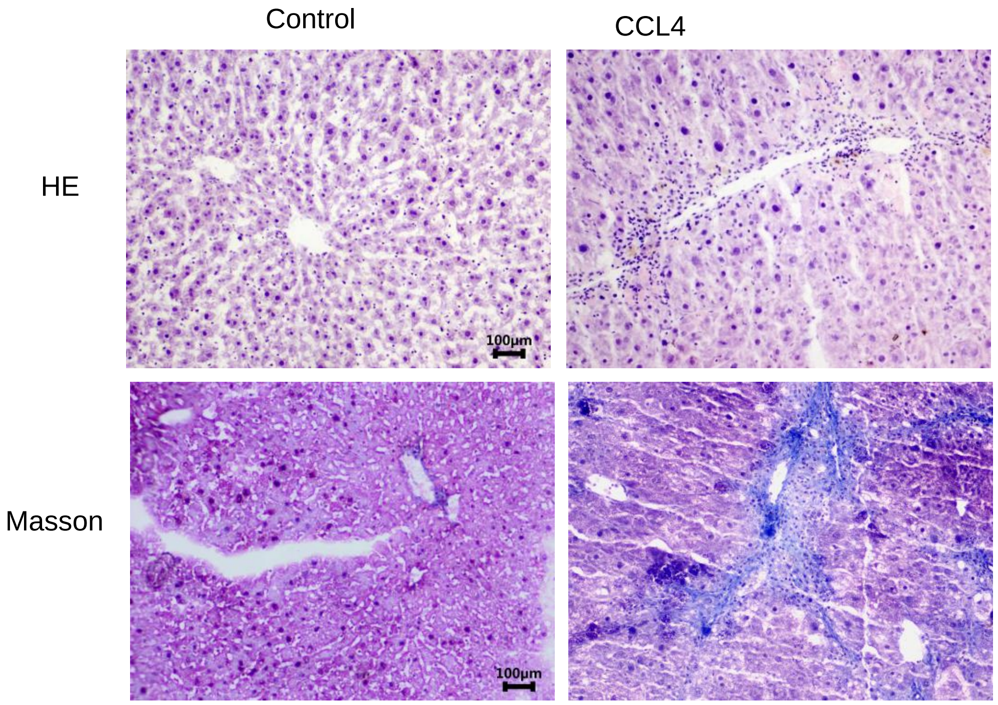

Unedited original Masson diagram for Figure 9A Masson assay was used to appraise collagen deposition in fibrotic liver tissues overexpressing miRNA-125a-5p

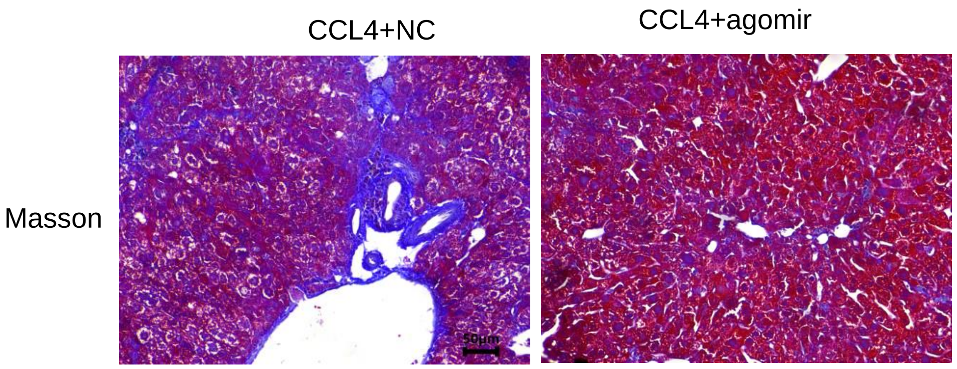

Supplement: Supplementary file 1 — The original diagram of HE+Masson [file 41420_2025_2694_MOESM1_ESM.pdf]
