# Supplementary material for: Overexpression miR-125a-5p inhibits HSCs activation and alleviates liver fibrosis through TGF-β/Smad2/3 signaling pathway and autophagy
Source: Cell Death Discov. 2025 Sep 1;11:419. doi: 10.1038/s41420-025-02694-4 (PMC12402229; doi:10.1038/s41420-025-02694-4)

Unedited original IHC diagram for Figure 6H immunohistochemistry was employed to evaluate TGFβR1 expression inside liver tissues in liver fibrosis mice overexpressing miRNA-125a-5p

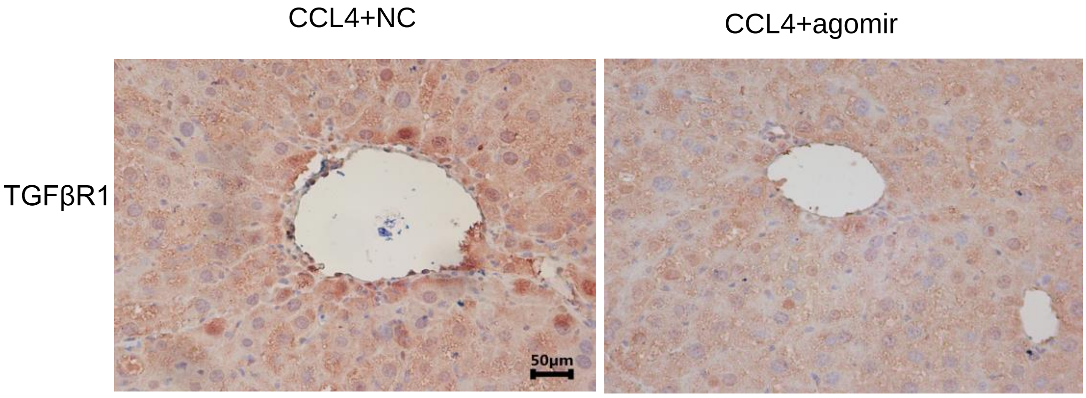

Unedited original IHC diagram for Figure 7B immunohistochemistry was used to detect α-SMA and Collagen Ⅰ levels in fibrotic liver tissues

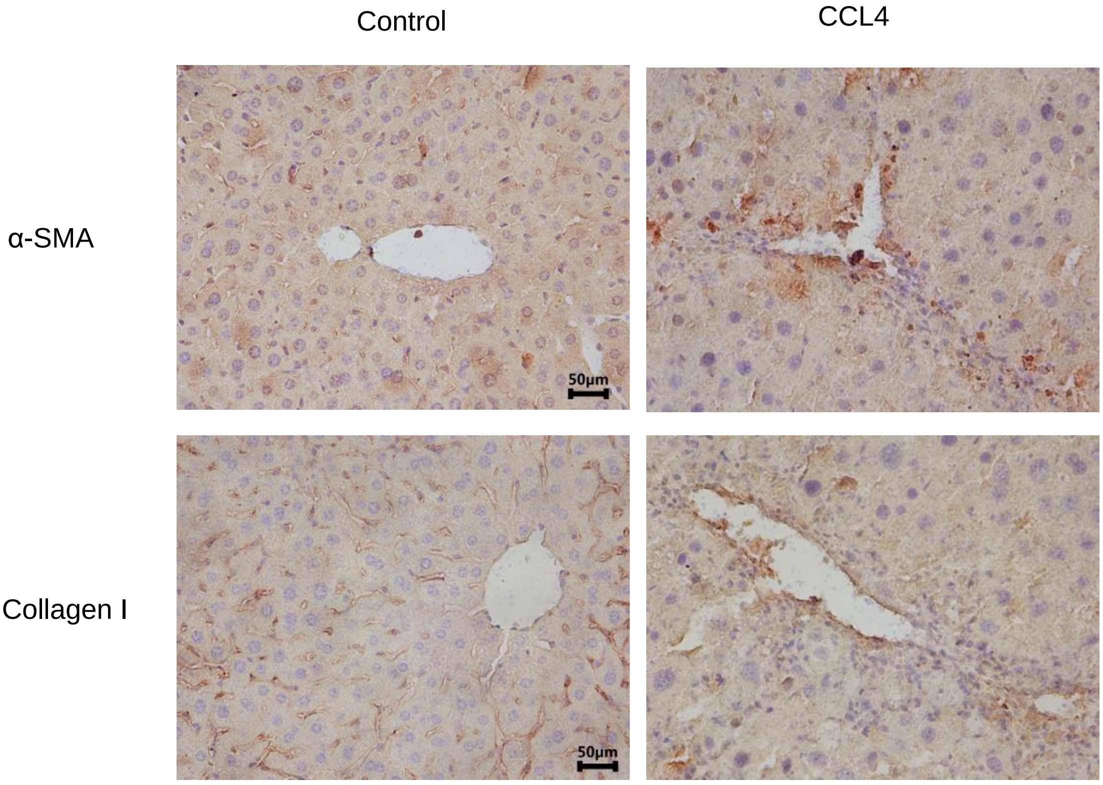

Unedited original IHC diagram for Figure 9D immunohistochemistry was used to detect the α-SMA and Collagen Ⅰ expression within fibrotic mouse liver tissues overexpressing miRNA-125a-5p

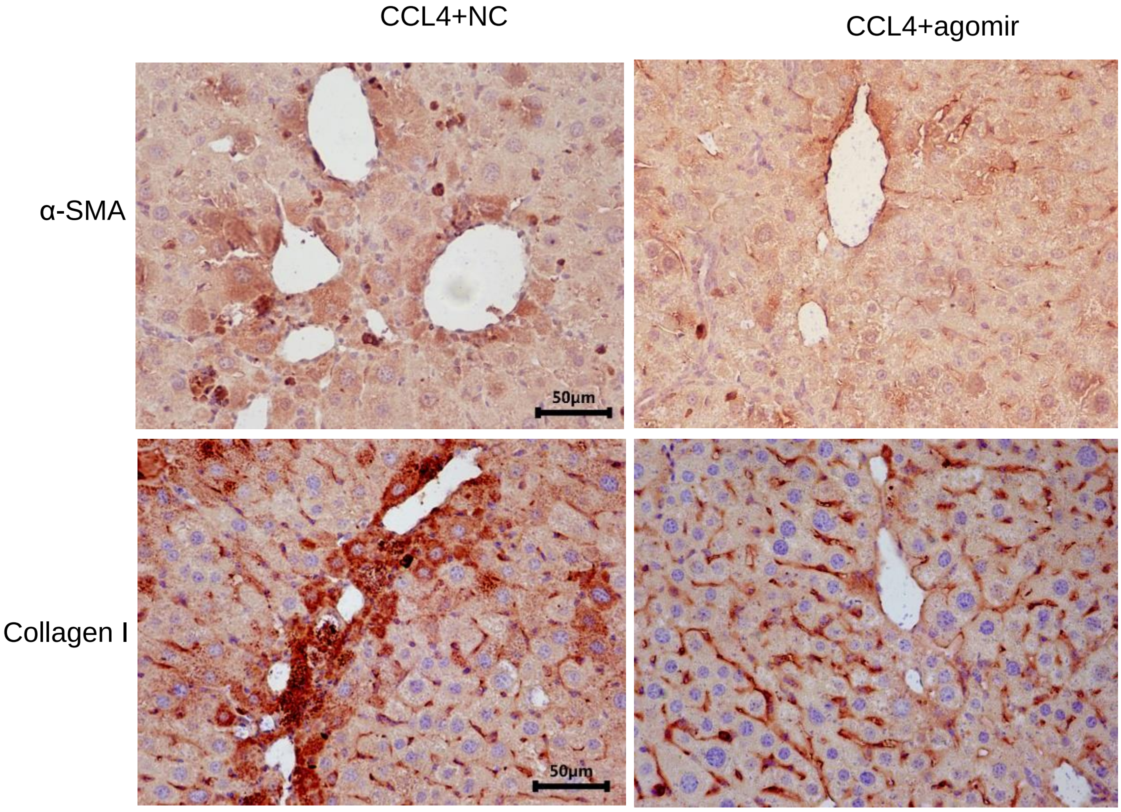

Unedited original IHC diagram for Figure 10D IHC was used to detectthe key proteins of autophagy and TGF-β/Smad pathway in fibrotic mouse liver tissues overexpressing miRNA-125a-5p

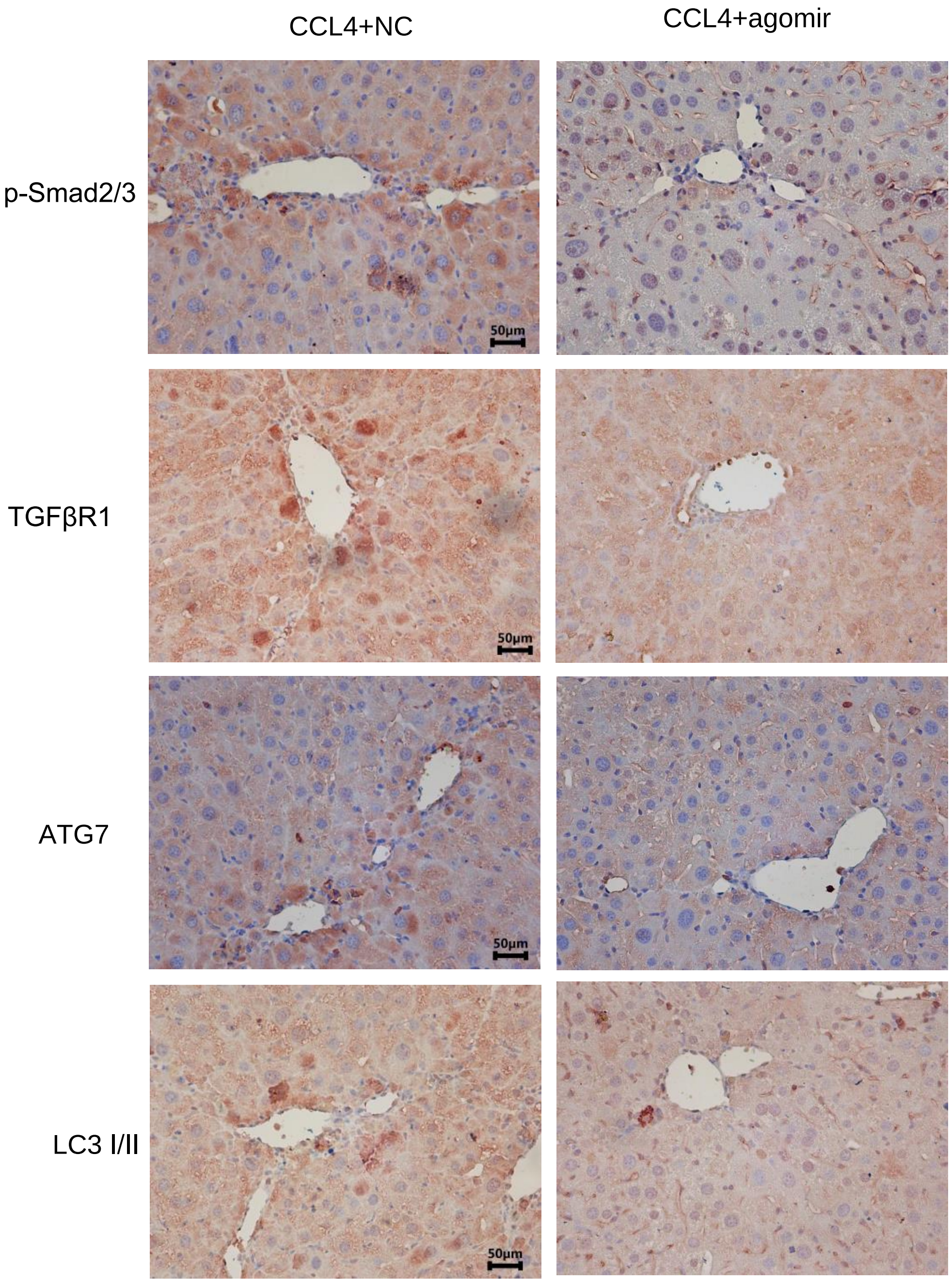

Supplement: Supplementary file 2 — The original diagram of IHC [file 41420_2025_2694_MOESM2_ESM.pdf]
