# Supplementary material for: Overexpression miR-125a-5p inhibits HSCs activation and alleviates liver fibrosis through TGF-β/Smad2/3 signaling pathway and autophagy
Source: Cell Death Discov. 2025 Sep 1;11:419. doi: 10.1038/s41420-025-02694-4 (PMC12402229; doi:10.1038/s41420-025-02694-4)

Unedited original IF diagram for Figure 1A immunofluorescence was carried out for evaluating α-SMA and Collagen Ⅰ levels in activated HSC-T6

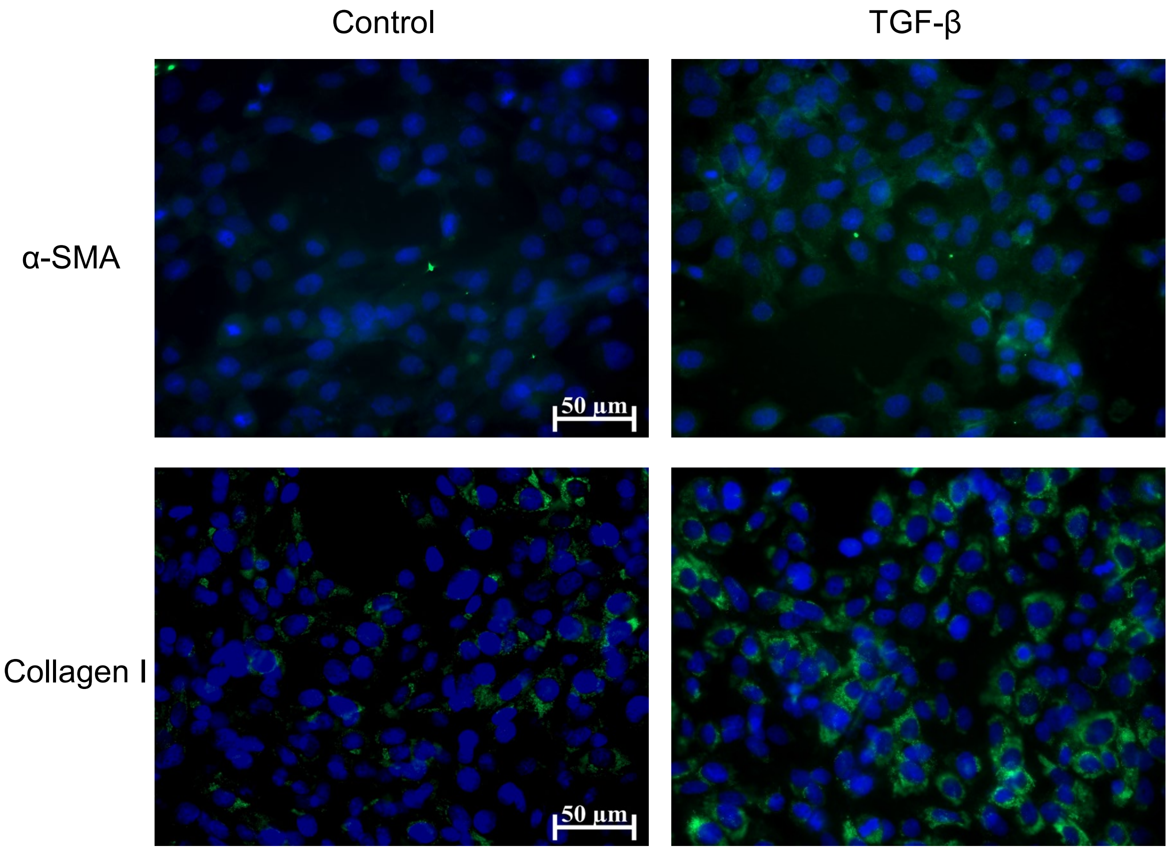

Unedited original IF diagram for Figure 2E immunofluorescence was employed to measurethe α-SMA and Collagen Ⅰ levels in activated HSC-T6 overexpression miR-125a-5p

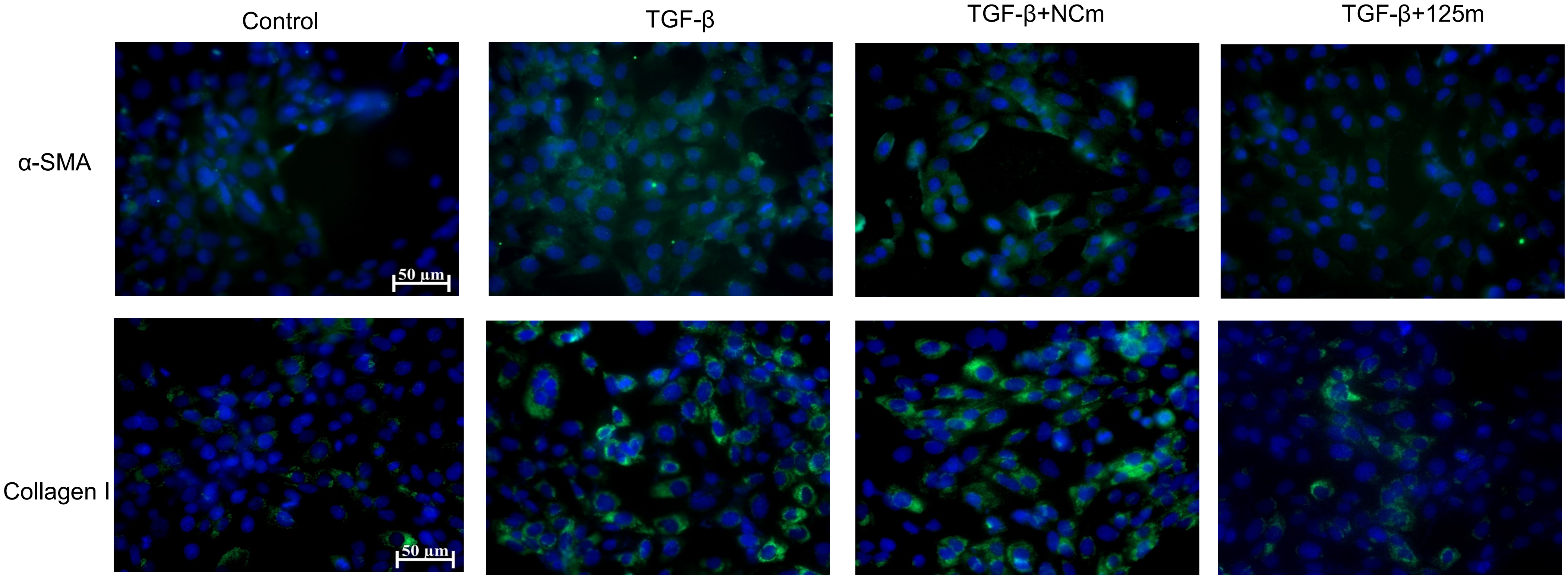

Unedited original IF diagram for Figure 3D immunofluorescence was employed to measure LC3 and ATG7 expression in activated HSC-T6 overexpression miR-125a-5p

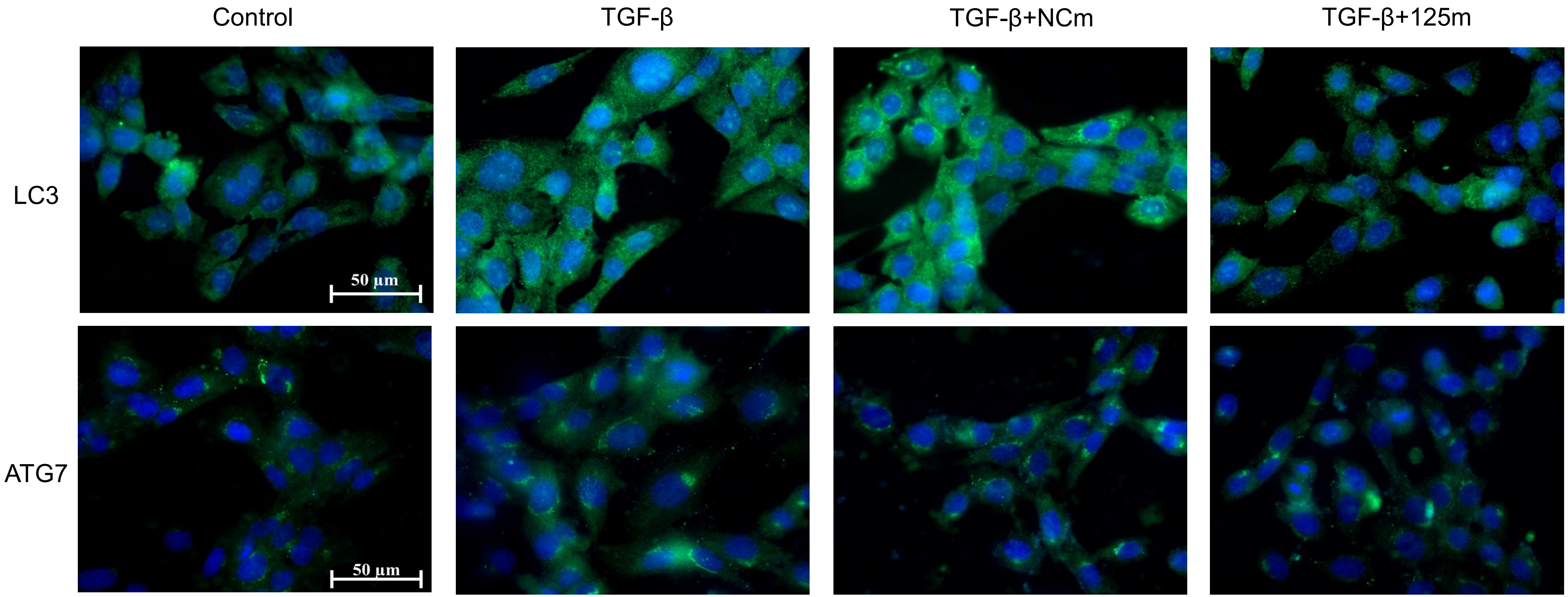

Unedited original IF diagram for Figure 4C immunofluorescence was employed to measure the expression of TGFβR1 and p-Smad2/3 in activated HSC-T6 overexpression miR-125a-5p

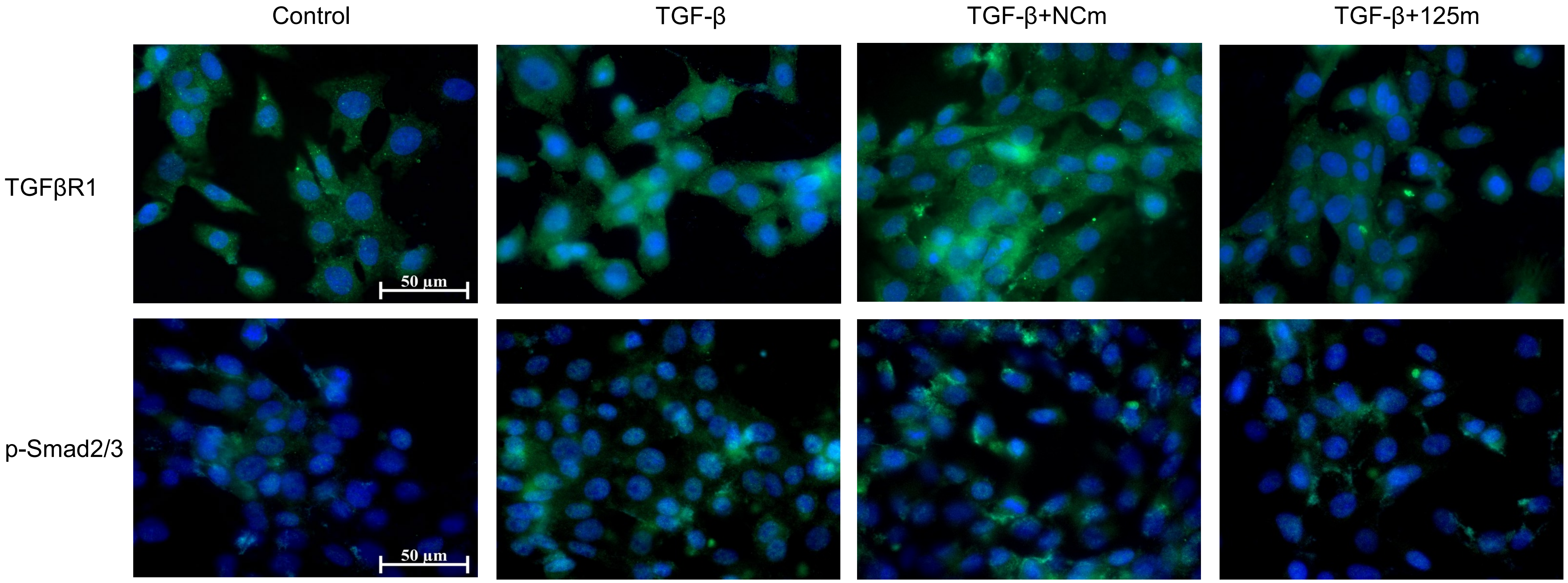

Unedited original IF diagram for Figure 6E immunofluorescence was used to evaluate TGFβR1 expression in HSC-T6 cells overexpressing miRNA-125a-5p

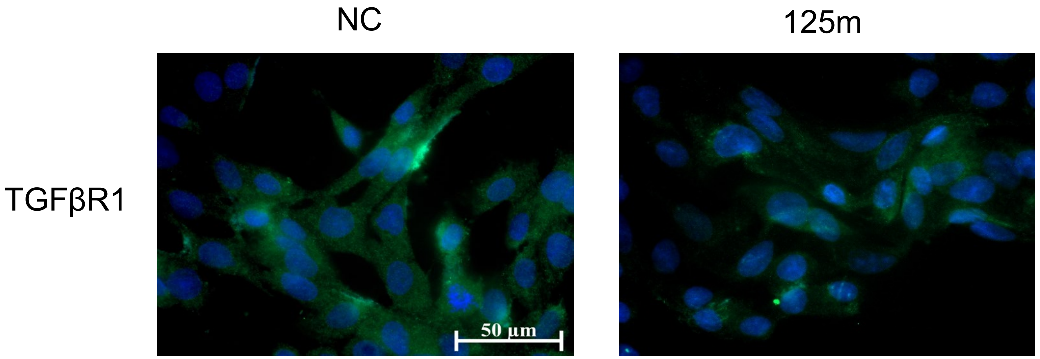

Unedited original fluorescence diagram for Figure 8A fluorescence microscope was employed for detecting the miRNA-125a-5p agomir transfection efficiency in mice with liver fibrosis

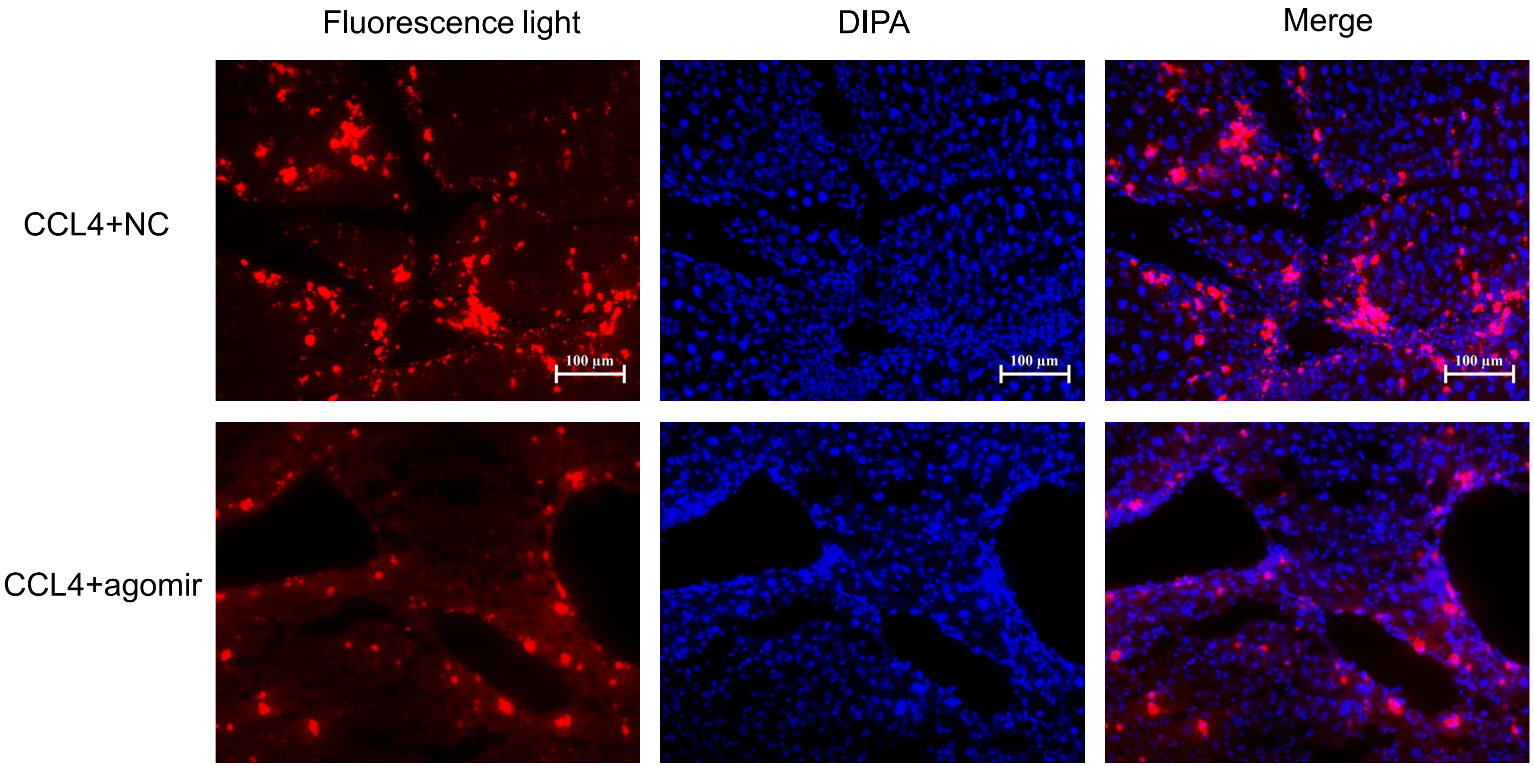

Supplement: Supplementary file 3 — The original diagram of immunofluorescence [file 41420_2025_2694_MOESM3_ESM.pdf]
