# Supplementary material for: Overexpression miR-125a-5p inhibits HSCs activation and alleviates liver fibrosis through TGF-β/Smad2/3 signaling pathway and autophagy
Source: Cell Death Discov. 2025 Sep 1;11:419. doi: 10.1038/s41420-025-02694-4 (PMC12402229; doi:10.1038/s41420-025-02694-4)

Unedited original gel diagram for Figure1-B WB was conducted to evaluate α-SMA and Collagen Ⅰ protein levels

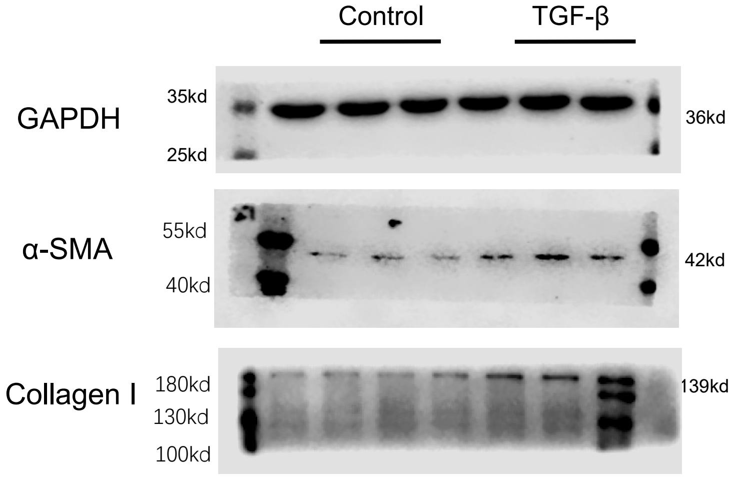

Unedited original gel diagram for Figure1-B WB was conducted to evaluate α-SMA and Collagen Ⅰ protein levels

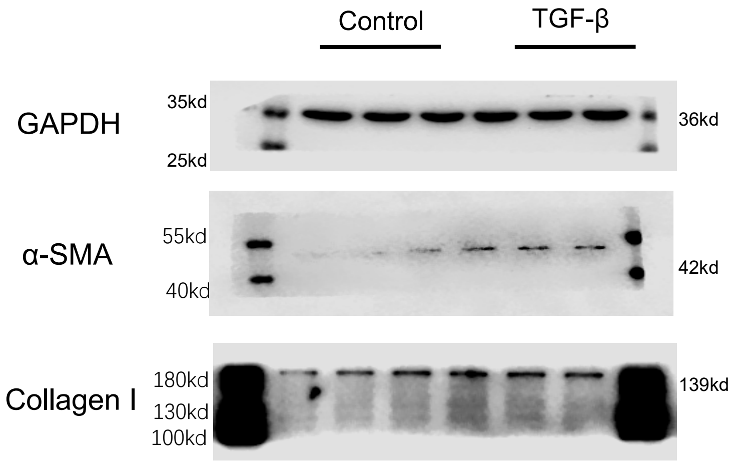

Unedited original gel diagram for Figure1-B WB was conducted to evaluate α-SMA and Collagen Ⅰ protein levels

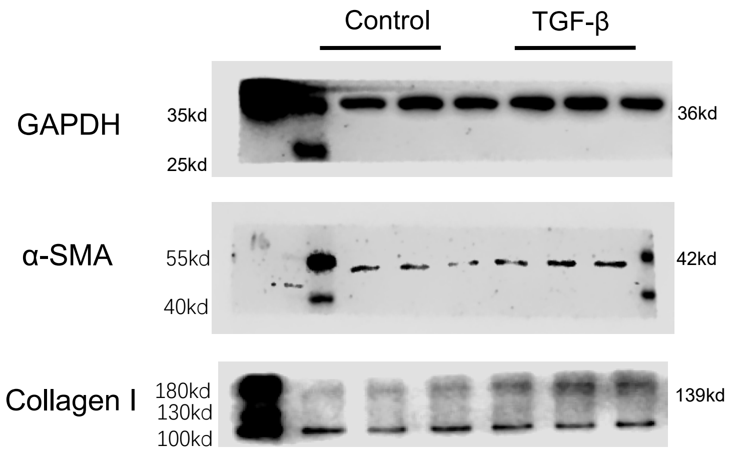

Unedited original gel diagram for Figure 2-C WB was performed for detecting α-SMA and Collagen Ⅰ protein levels

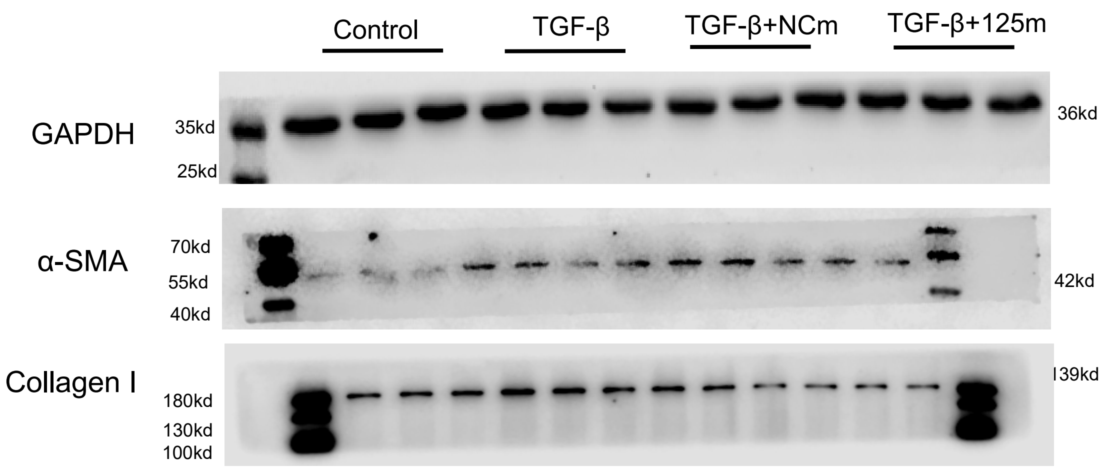

Unedited original gel diagram for Figure 2-C WB was performed for detecting α-SMA and Collagen Ⅰ protein levels

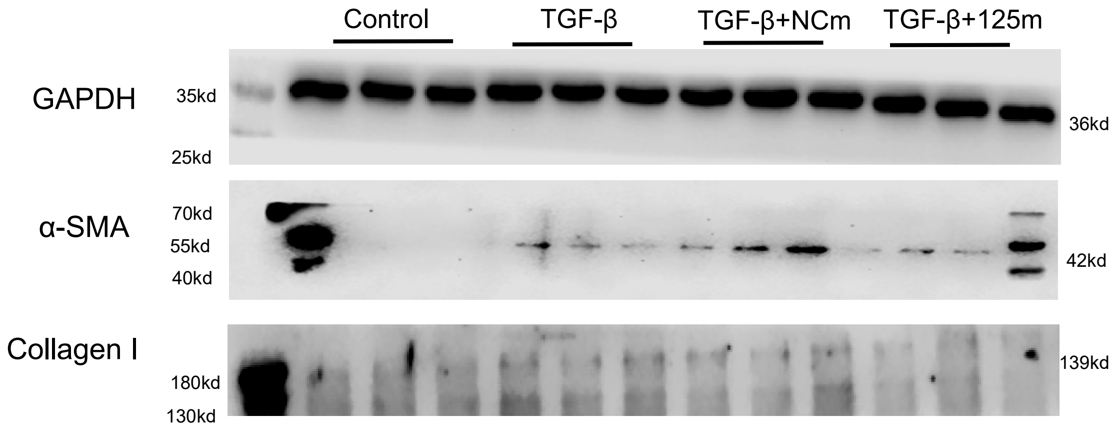

Unedited original gel diagram for Figure 2-C WB was performed for detecting α-SMA and Collagen Ⅰ protein levels

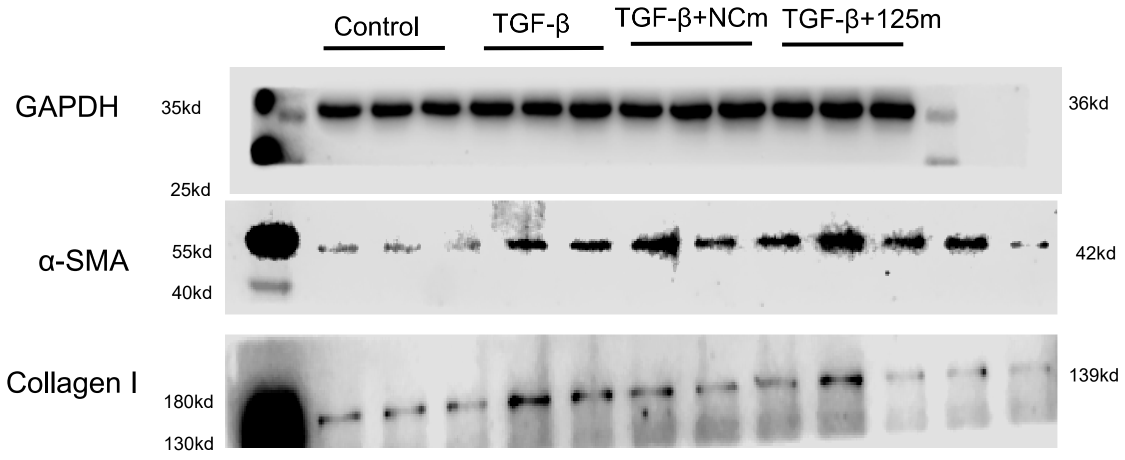

Unedited original gel diagram for Figure 3-A WB was conducted for evaluating LC3 and ATG7 protein levels

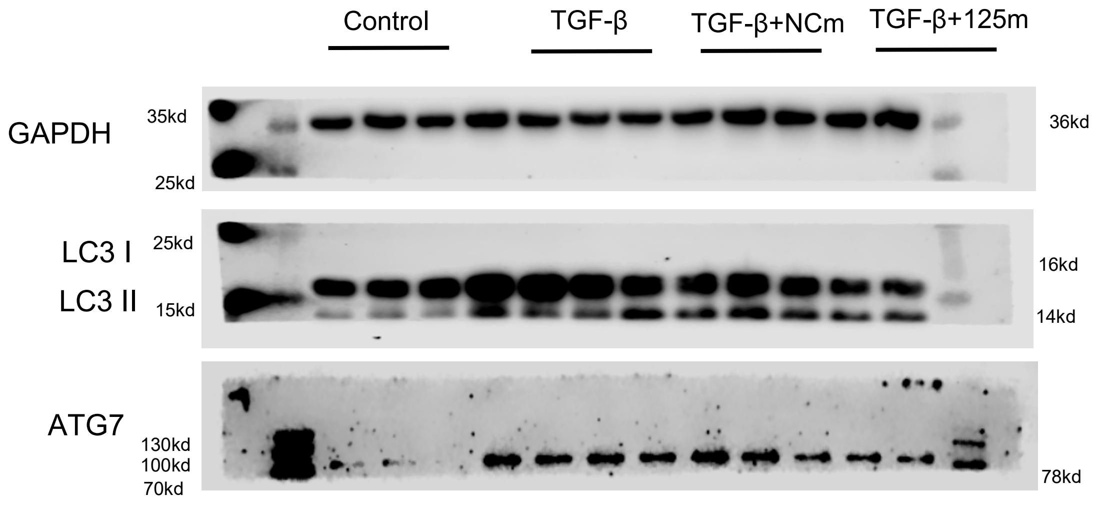

Unedited original gel diagram for Figure 3-A WB was conducted for evaluating LC3 and ATG7 protein levels

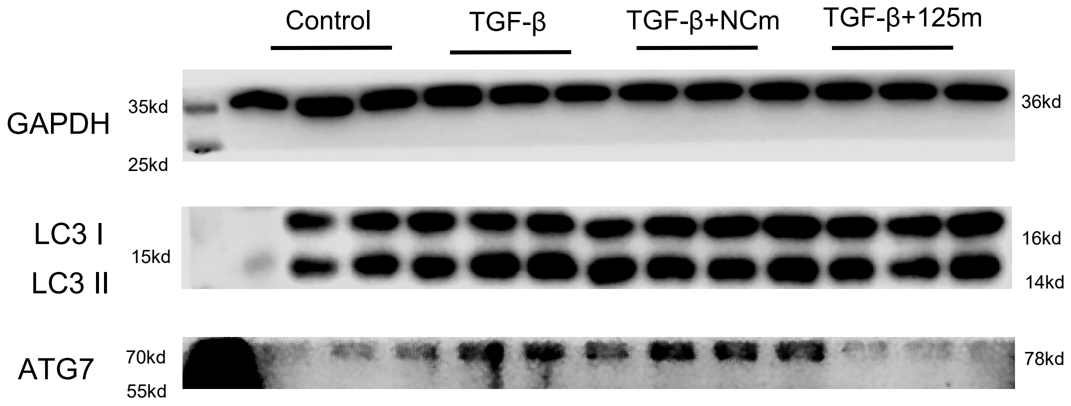

Unedited original gel diagram for Figure 3-A WB was conducted for evaluating LC3 and ATG7 protein levels

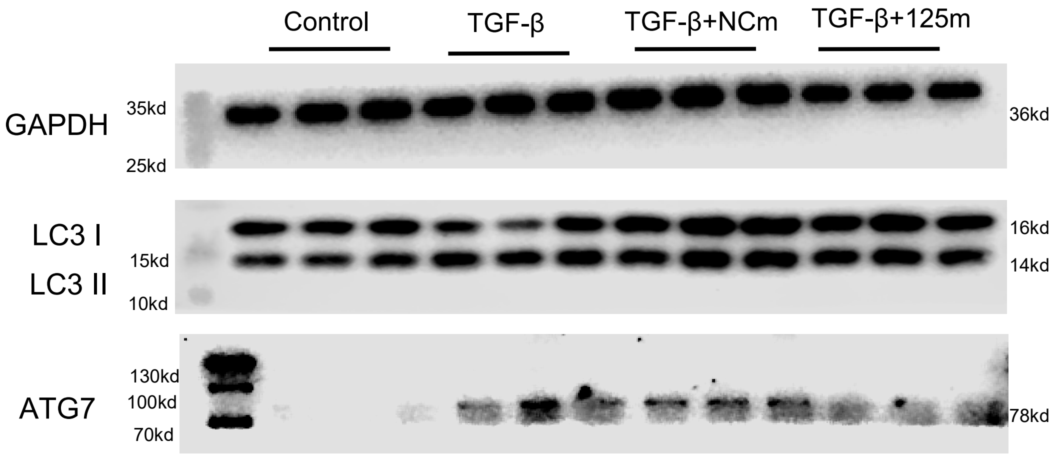

Unedited original gel diagram for Figure 4-A WB was conducted to estimate TGF-β, TGFβR1 and p-Smad2/3 levels

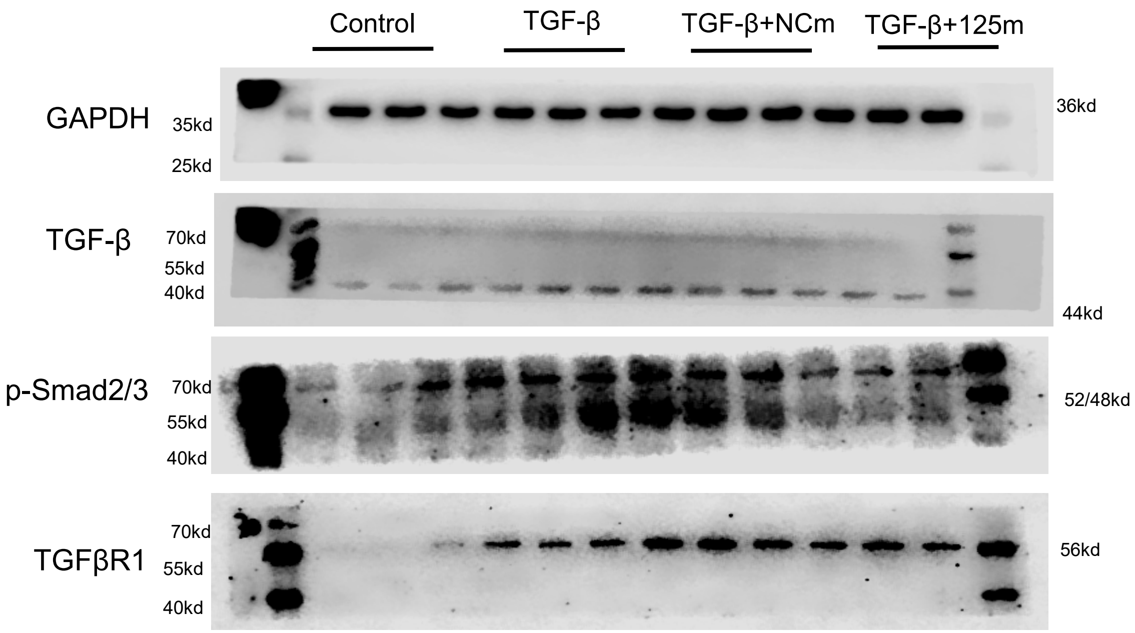

Unedited original gel diagram for Figure 4-A WB was conducted to estimate TGF-β, TGFβR1 and p-Smad2/3 levels

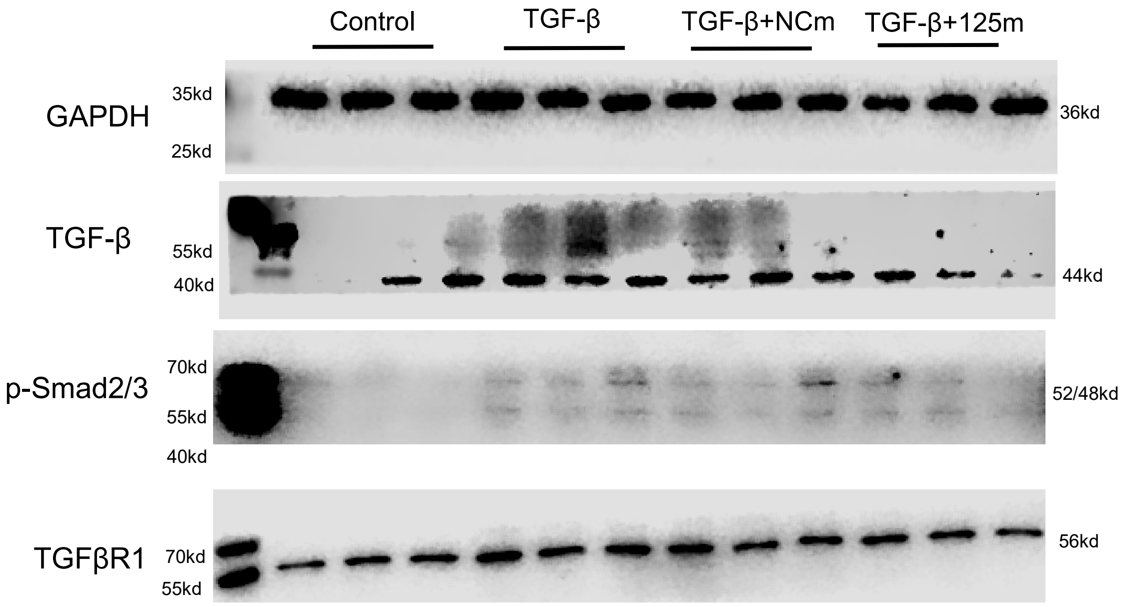

Unedited original gel diagram for Figure 4-A WB was conducted to estimate TGF-β, TGFβR1 and p-Smad2/3 levels

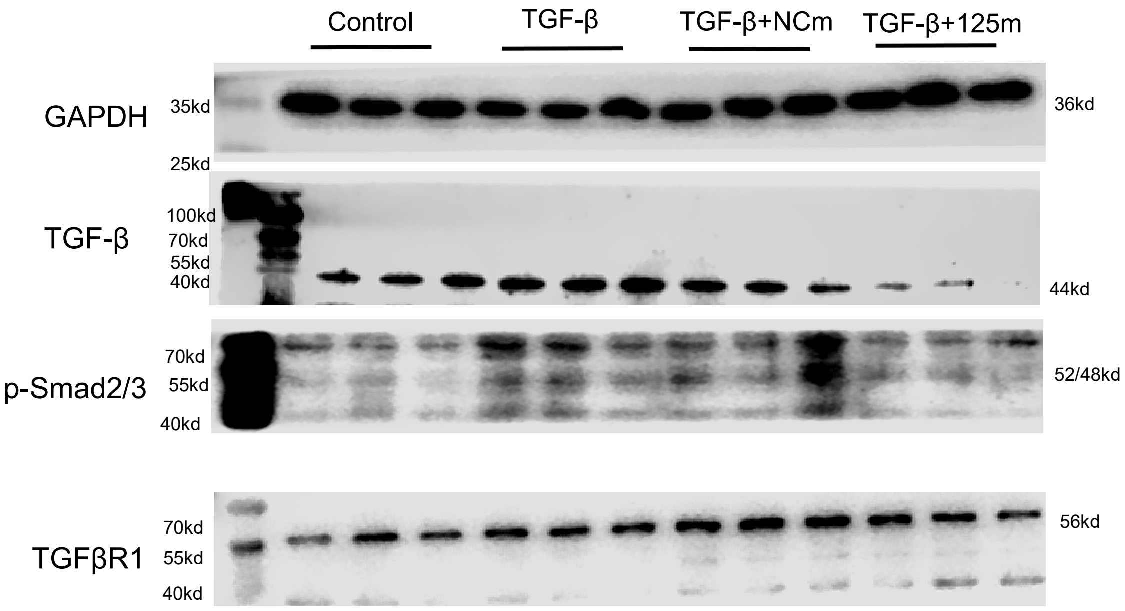

Unedited original gel diagram for Figure 6-C WB was performed for exploring TGFβR1 level in HSC-T6 and BRL-3A cells overexpressing miRNA-125a-5p

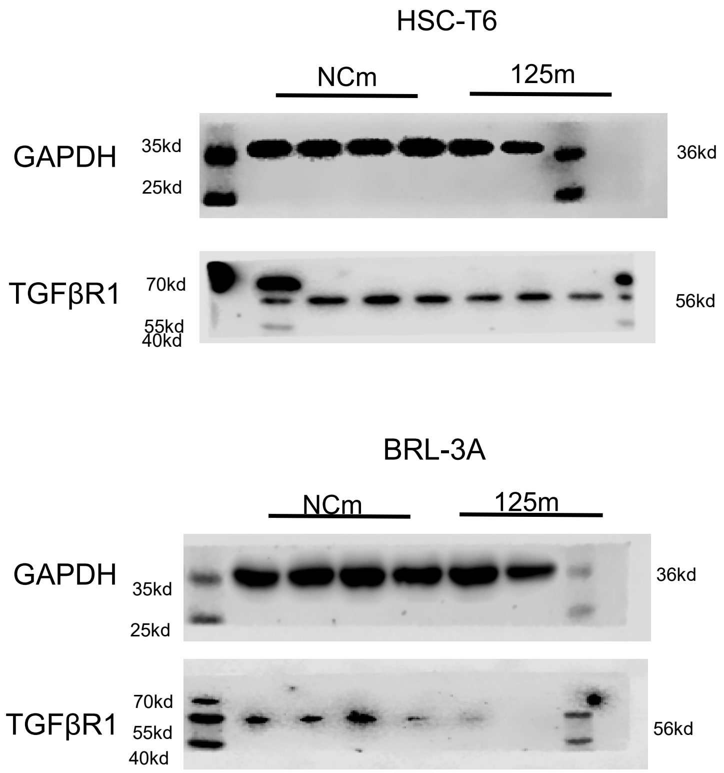

Unedited original gel diagram for Figure 6-C WB was performed for exploring TGFβR1 level in HSC-T6 and BRL-3A cells overexpressing miRNA-125a-5p

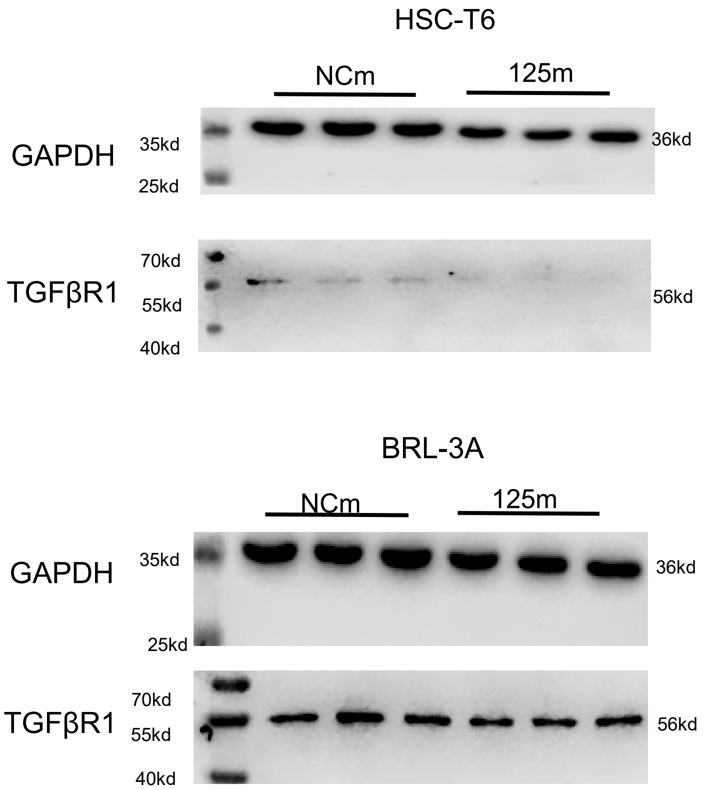

Unedited original gel diagram for Figure 6-C WB was performed for exploring TGFβR1 level in HSC-T6 and BRL-3A cells overexpressing miRNA-125a-5p

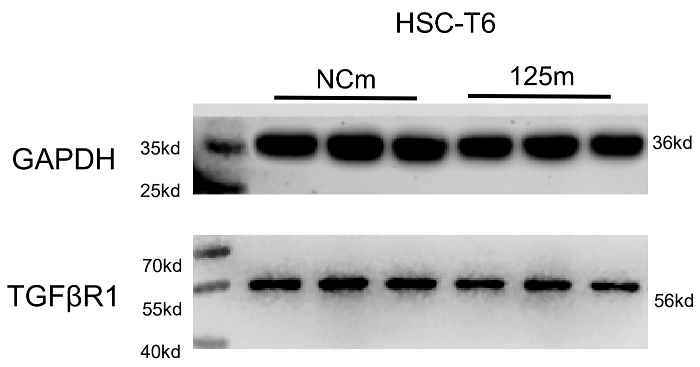

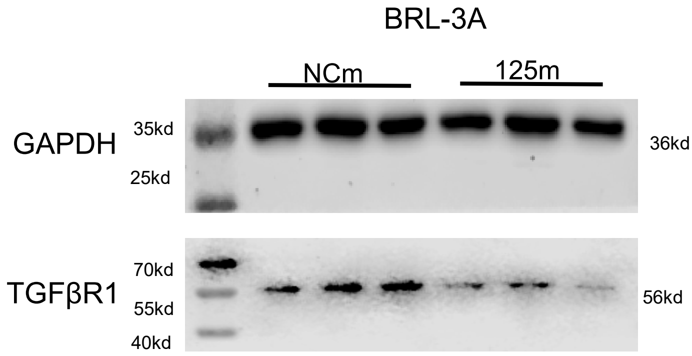

Unedited original gel diagram for Figure 6-F WB was conducted detect TGFβR1 expression in mouse liver tissues overexpressing miRNA-125a-5p

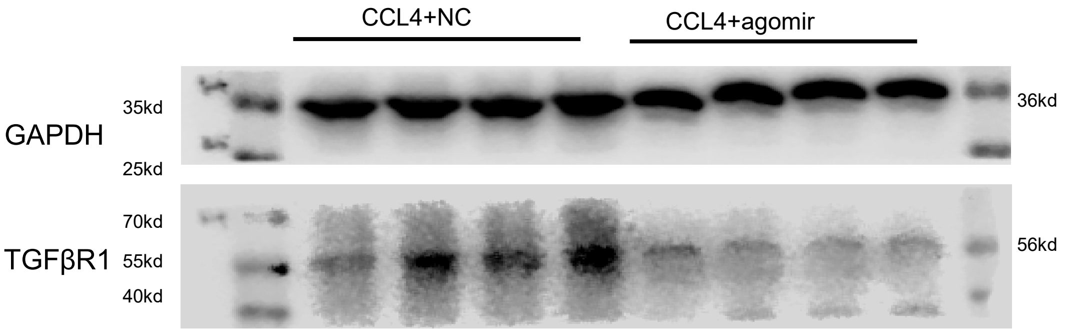

Unedited original gel diagram for Figure 6-F WB was conducted detect TGFβR1 expression in mouse liver tissues overexpressing miRNA-125a-5p

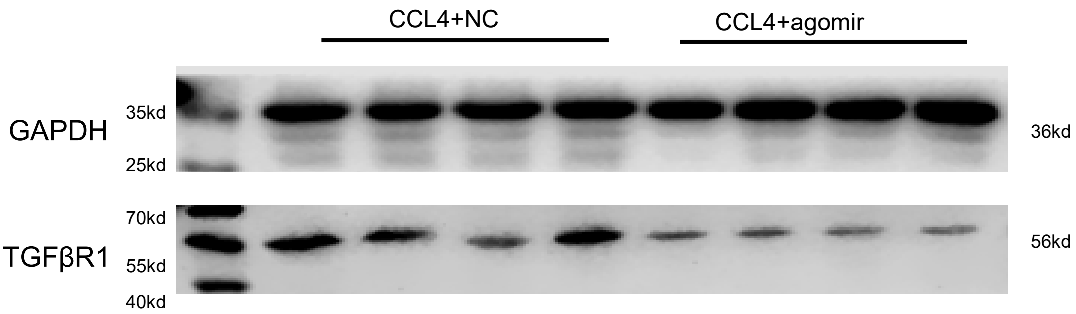

Unedited original gel diagram for Figure 6-F WB was conducted detect TGFβR1 expression in mouse liver tissues overexpressing miRNA-125a-5p

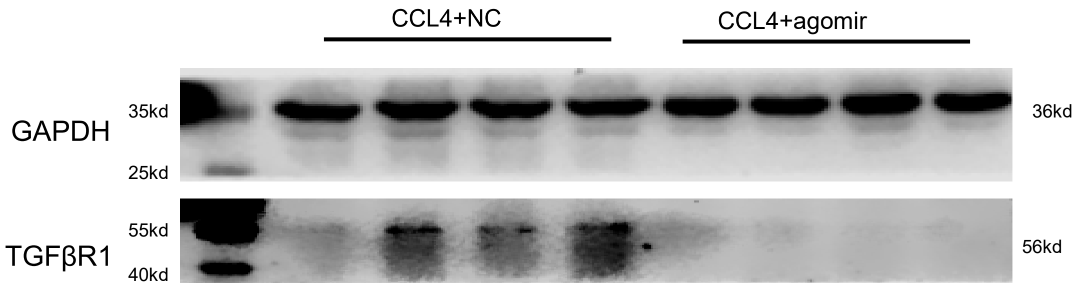

Unedited original gel diagram for Figure 7-C WB was performed to detect α-SMA and Collagen Ⅰ levels in fibrotic liver tissues

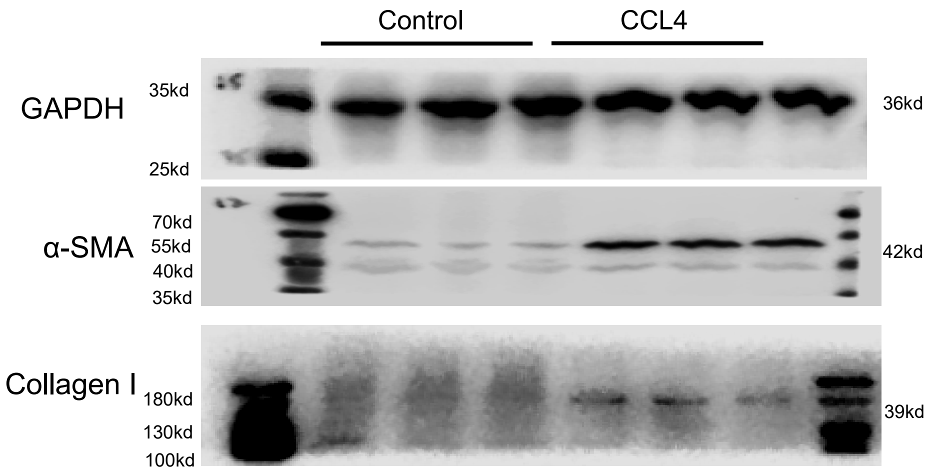

Unedited original gel diagram for Figure 7-C WB was performed to detect α-SMA and Collagen Ⅰ levels in fibrotic liver tissues

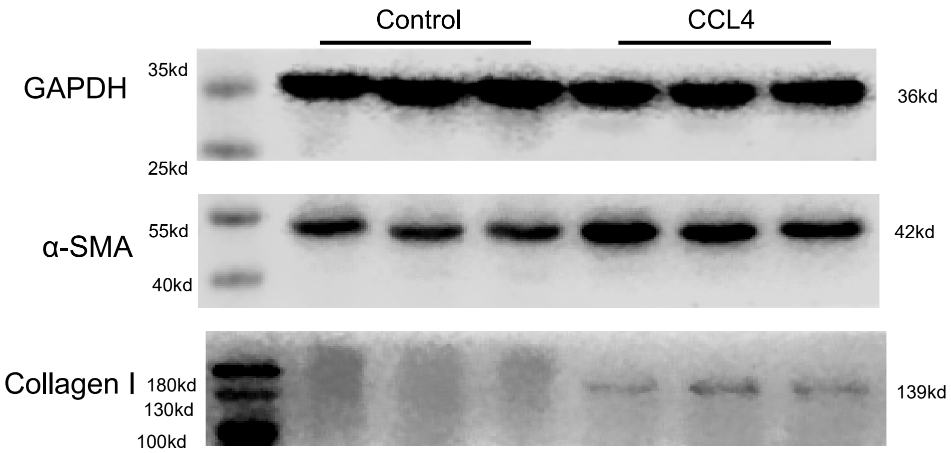

Unedited original gel diagram for Figure 7-C WB was performed to detect α-SMA and Collagen Ⅰ levels in fibrotic liver tissues

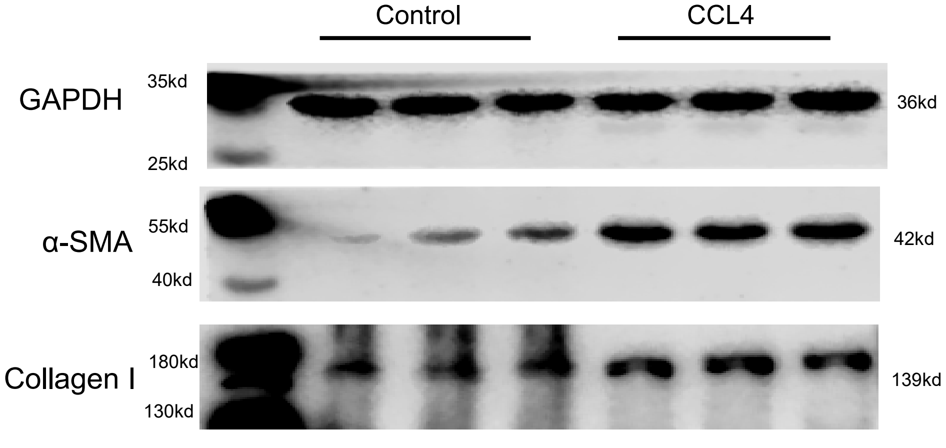

Unedited original gel diagram for Figure 9-B WB was conducted to evaluate the α-SMA and Collagen Ⅰ levels in fibrotic liver tissues overexpressing miRNA-125a-5p

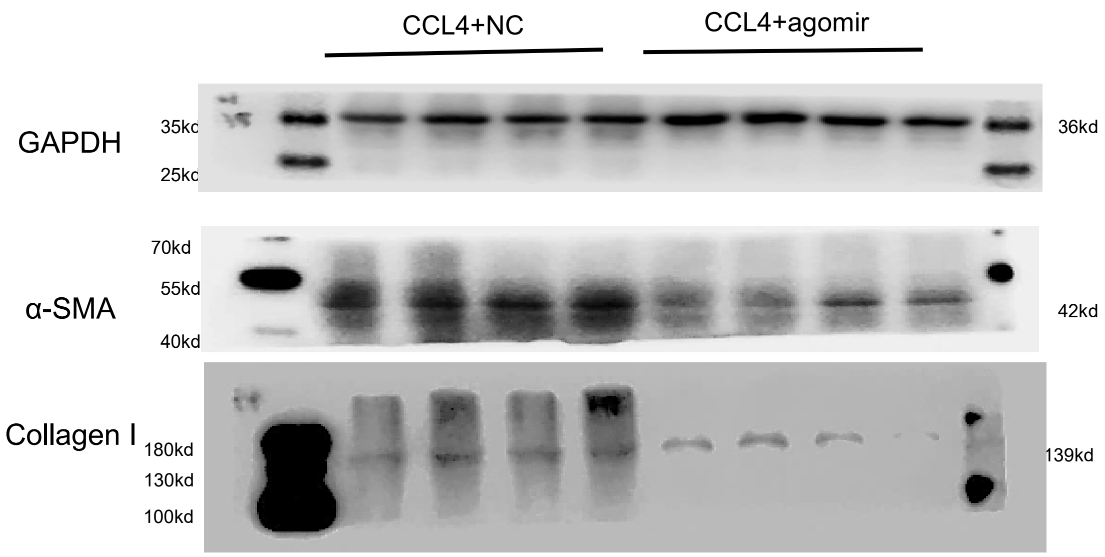

Unedited original gel diagram for Figure 9-B WB was conducted to evaluate the α-SMA and Collagen Ⅰ levels in fibrotic liver tissues overexpressing miRNA-125a-5p

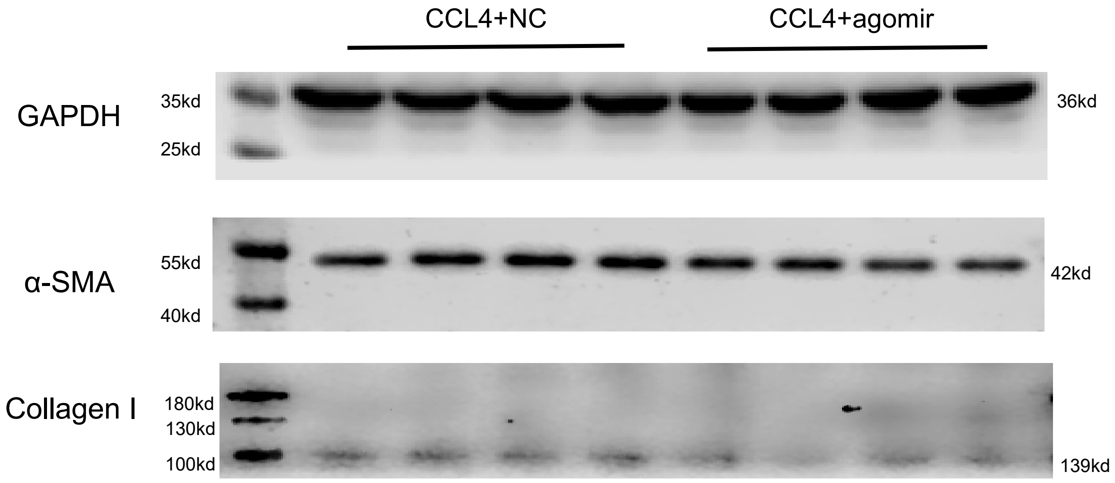

Unedited original gel diagram for Figure 9-B WB was conducted to evaluate the α-SMA and Collagen Ⅰ levels in fibrotic liver tissues overexpressing miRNA-125a-5p

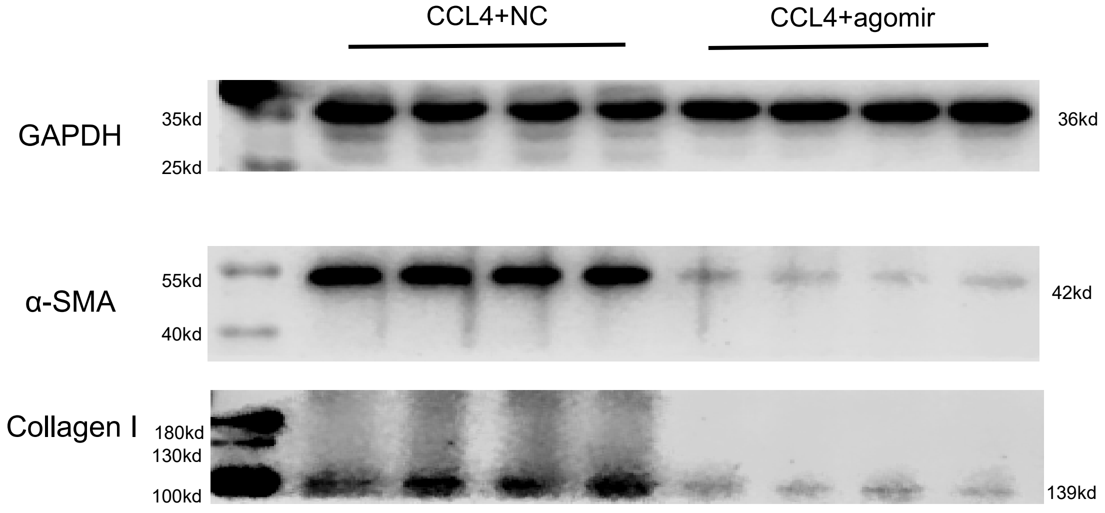

Unedited original gel diagram for Figure 10-A WB was conducted to evaluate the key proteins of autophagy and TGF-β/Smad pathway in mouse liver tissues overexpressing miRNA-125a-5p

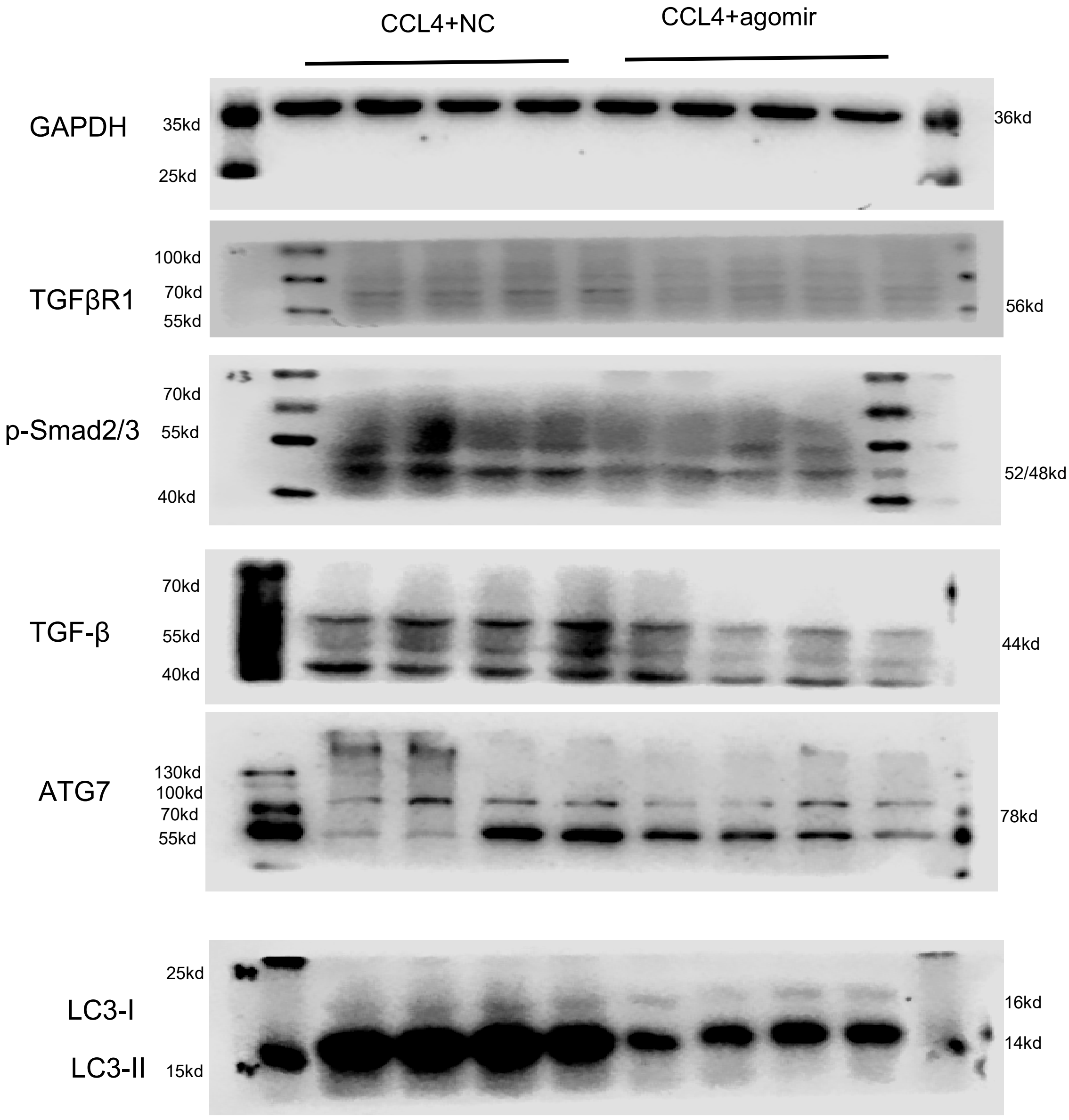

Unedited original gel diagram for Figure 10-A WB was conducted to evaluate the key proteins of autophagy and TGF-β/Smad pathway in mouse liver tissues overexpressing miRNA-125a-5p

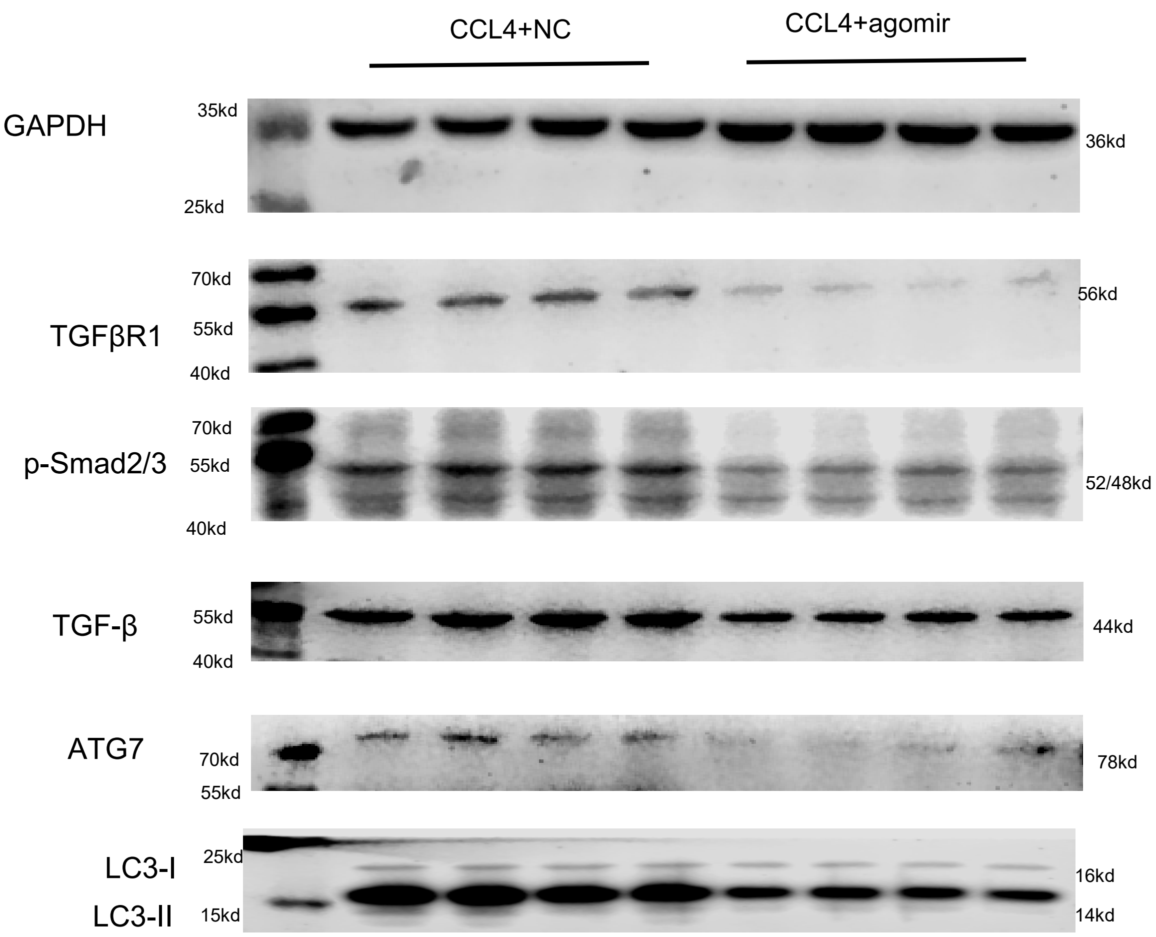

Unedited original gel diagram for Figure 10-A WB was conducted to evaluate the key proteins of autophagy and TGF-β/Smad pathway in mouse liver tissues overexpressing miRNA-125a-5p

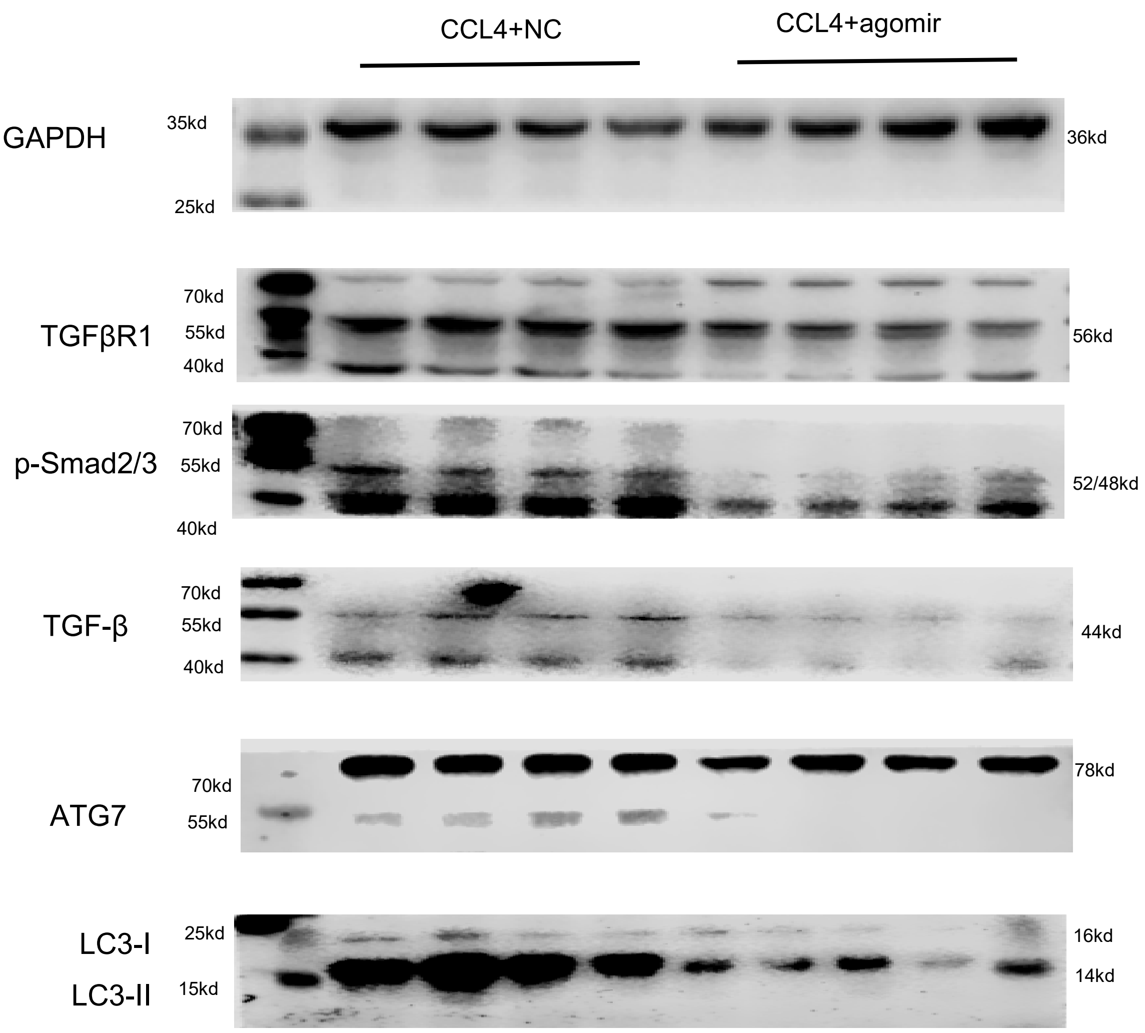

Supplement: Supplementary file 4 — The original gel diagram of WB [file 41420_2025_2694_MOESM4_ESM.pdf]
